# Preferences of nursing and medical students for working with older adults and people with dementia: a systematic review

**DOI:** 10.1186/s12909-020-02000-z

**Published:** 2020-03-30

**Authors:** Molly Hebditch, Stephanie Daley, Juliet Wright, Gina Sherlock, James Scott, Sube Banerjee

**Affiliations:** 1 Centre for Dementia Studies, Brighton & Sussex Medical School, University of Sussex, Falmer, BN1 9RY UK; 2 Department of Medical Education, Brighton and Sussex Medical School, Falmer, BN1 9RY UK; 3 Faculty of Health, University of Plymouth, Devon, PL4 8AA UK

**Keywords:** Dementia, Healthcare students, Career preferences

## Abstract

**Background:**

A current issue in workforce planning is ensuring healthcare professionals are both competent and willing to work with older adults with complex needs. This includes dementia care, which is widely recognised as a priority. Yet research suggests that working with older people is unattractive to undergraduate healthcare students.

**Methods:**

The aim of this systematic review and narrative synthesis is to explore the factors related to healthcare (medical and nursing) student preferences’ for working with older people and people with dementia. Searches were conducted in five databases: MEDLINE, PsycINFO, CINHAL, BNI, ERIC. Screening, data extraction and quality appraisal were conducted by two independent reviewers. A narrative, data-based convergent synthesis was conducted.

**Results:**

One thousand twenty-four papers were screened (139 full texts) and 62 papers were included for a narrative synthesis. Factors were grouped into seven categories; student characteristics, experiences of students, course characteristics, career characteristics, patient characteristics, work characteristics and the theory of planned behaviour.

**Conclusion:**

Health educators should review their role in cultivating student interest in working with older adults, with consideration of student preparation and the perceived value of this work. There is a lack of evidence about the career preferences of students in relation to dementia, and this warrants further research.

## Background

The demographics of the world’s population are changing, as people are living longer [1, 2]. The impact on healthcare is pervasive. Older people are frequent users of healthcare services with high multi-morbidly [3, 4]. Healthcare professionals are likely to work with older adults regardless of their speciality or healthcare setting. Increasing the capacity of the healthcare workforce to provide competent and effective care for older adults is an international concern [2]. This includes the need for more specialists in the care of the elderly, such as geriatricians and gerontological nurses [5, 6] as well as generalists with adequate skills in older adult care [2, 7]. Despite this need, working with older adults and the associated specialities is consistently documented as unpopular with healthcare students [8–13]. Career preferences formed during training can be predictive of future career choices and behaviour in practice [14, 15]. Therefore, it is important to understand the factors that may influence student preferences for consideration in education and workforce planning.

Previous systematic reviews have explored the factors associated with a low preference of working with older adults in nursing students [16–18] and geriatrics in medical students [19]. However, no reviews have included papers of both nursing and medical students allowing direct comparisons to be made. Also, previous reviews have only included preferences for either geriatrics or long-term care settings and excluded studies of educational interventions. Finally, none have included preferences for working with people with dementia where education and care practices are internationally recognised as suboptimal [20–22]. This review therefore sought to explore comprehensively the potential factors related to preferences of healthcare students in relation to working with older people and people with dementia using broad criteria for these preferences, comprising any specialities, settings or patient populations related to older people.

## Method

A protocol was written adhering to PRISMA-P guidelines [23] and registered on The International Prospective Register of Systematic Reviews-CRD42018104647 [24].

### Eligibility criteria

A summary of the eligibilty criteria is presented in Table 1.

**Table 1:** Summary of Eligibility Criteria

**Inclusion**	
1. Career preferences AND 2. Older adults OR dementia, AND 3. Medical OR Nursing students	
Topics:	
• Factors associated with career preferences OR • Career preferences as an outcome of an educational intervention	
**Exclude**	
• Postgraduate training or registered healthcare professionals (up to specialist training for medical) • Career preferences not related to either older adults in general or dementia	

#### Population

The population of interest included medical and nursing students and excluded all other healthcare disciplines. Studies that involved an additional student group were excluded unless findings were separately identified for medical and/or nursing students.

#### Construct of interest

The construct of interest was student preferences for working with older adults or people with dementia. Due to variability in terms, this included measures of ‘intent to work’, ‘career choices’ or in medical students ‘speciality choice or interest’. There was no restriction on the type of measures of career preferences. Qualitative explorations of preferences were included. The types of preference measured required direct relevance to older adults or dementia. This included preferences measured in relation to patient populations, specialities and settings associated with older adults or dementia.

#### Types of studies

All empirical articles were included, including quantitative, qualitative and mixed-method studies and theses. Conference reports or opinion pieces were excluded. Studies must have been published in English. Only studies published from 1995 onwards were included. Studies explored career preferences with associated factors or educational interventions. If a study only explored the relationship between an intervention and career preferences, a comparison group was required for inclusion.

### Information sources

The initial search was conducted on the 20th September 2018 on the following databases: MEDLINE, PsycINFO, CINHAL, BNI, ERIC and google scholar. To identify further possible relevant articles, the references of included articles and relevant systematic reviews were searched.

### Search strategy

Initial search terms were formed during scoping exercises. A specialist librarian was consulted to inform the final search strategy. Key terms included: ((preference adj3 work*)“ “career preference” or “career choice” or “intent* to work” or speciali*ation or “career intent*” or “special*ty choice” or “special*ty interest”) AND (“older adult*” or “older people” or elder* or dementia or geriatric* or aged) AND (student* ad j3 nurs* or “medical student*” or “allied health* student*” or “health* student*). Index terms (e.g MeSH) were also used alongside these key terms. An example search is included in Additional file 1.

### Study selection

Identified references were added to EndNote (version X7). After duplicates were removed, articles were screened against eligibility criteria independently by two reviewers (MH & JS) by title and abstract, and then by full text. If there was disagreement, a third reviewer was consulted (SD).

### Data extraction and analysis

An extraction template was developed and piloted by the reviewing team (MH, JS, GS, SD) and can be seen in Additional file 2. Only relevant data to preferences was extracted and only data related to nursing or medical students. Statistical probability set at < 0.05, or else recorded as non-significant. For qualitative studies: only major themes (with clear description/quotes), clearly related to preferences, were included as factors.

Papers were extracted independently by two reviewers; MH and either GS and JS, with disagreements resolved by SD. Papers that presented results from the same study were consolidated, to avoid conflation of results.

### Quality assessment

Risk of bias was explored using the Mixed Methods Appraisal Tool (MMAT) scale [25], as it takes particular consideration of mixed-method studies [26]. Each paper was rated during extraction by two independent reviewers. Papers were not excluded based on MMAT scores due to the exploratory nature of this review. A narrative description of the overall quality is presented with MMAT scores.

### Synthesis

A narrative synthesis approach [27] was used as it allows integration of qualitative, quantitative and mixed-method studies and a quantitative meta-synthesis would not be possible due to the variability of definitions and measurements for career preferences. The use of mixed-method studies means that a data-based convergent synthesis was used [26]. Factors were identified by researchers inductively, using the most consistently used terms by the authors of papers where possible. The labelling of factors for both quantitative variables and qualitative themes were considered with the team to assess fit.

## Results

### Study selection

**Fig. 1:**
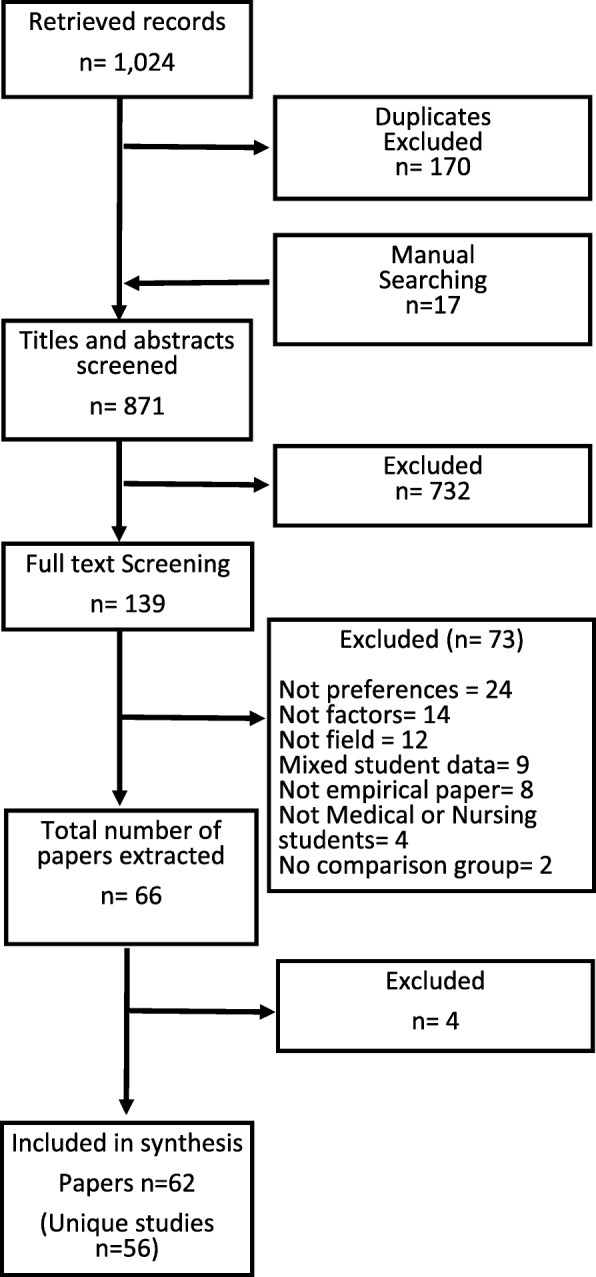
Flow Diagram of Study Selection

Figure 1 below outlines the number excluded at each stage.

Sixty-six papers were included for data extraction. Four studies were excluded during extraction because they did not meet the inclusion criteria. This comprised 56 unique studies (62 papers) after considering the multiple publication of data. An overview of each study is presented in Additional file 3.

### Studies characteristics

The majority of studies were cross-sectional in design (*n* = 30). There were nine quasi-experimental studies, seven qualitative, seven longitudinal studies and three mixed-method designs.

The highest number of studies was from the USA (*n* = 14) followed by Australia (*n* = 6), Canada (*n* = 5), UK (*n* = 5) and Israel and China (*n* = 3). There were two papers each from Hong Kong, Turkey, Taiwan, Sweden, Saudi Arabia, and one from Finland, Ireland, Jordon, New Zealand, Norway, Malaysia, Philippines, Singapore and Sri-Lanka. One paper compared results from Australia and China.

Thirty-eight studies investigated the preferences of nursing students, 17 of medical students, with only a single study exploring both.

#### Career preference definitions

Of the 18 studies that explored preferences of medical students, 16 investigated interest, willingness or likelihood of pursuing geriatrics, one explored preferences for working with older people and a single study looked at preferences towards working with people with dementia.

The type of career preferences explored for nursing students was varied and was often inconsistent within studies. Only one study investigated the intention to work in dementia care [28]. The majority of studies looked at preferences for working with older people.

#### Measurements of career preferences

The most common type of quantitative measure of preferences was a single item (*n* = 23). Eleven studies used a variation of a ranking scale, with the most common based on the work of Stevens and Crouch [29]. Unique scales were described in 14 studies, including one measure of dementia preference [28].

In the seven qualitative studies, methods of investigating preferences included focus groups (*n* = 4), individual interviews (*n* = 2) and a semi-structured questionnaire (*n* = 1). Other qualitative methods used included reflective essays and open text questions.

### Research quality

Research quality was variable. Out of a possible score of five on the MMAT, 6 studies scored two, 21 scored three, 22 scored four and 7 scored five. Individual scores are presented in Additional file 3. Consistent issues included the use of non-standardised measures and poor construct definition. Additionally, where educational interventions were evaluated, these often lacked control groups or did not allow for confounding variables. Finally, longitudinal studies had constantly low follow up rates (< 60% for follow up).

### Narrative synthesis

A summary of synthesised factors associated with preferences for working with older people or people with dementia can be seen in Additional file 4. Factors are represented by either quantitative variables or qualitative themes. These factors were grouped into seven categories, which are discussed as follows:

1. Student characteristics
2. Experiences of students
3. Course characteristics
4. Career characteristics
5. Patient characteristics
6. Work characteristics
7. Theory of planned behaviour

### Student characteristics

#### Demographics

There was support for a positive association of preference with female gender [9, 30–34]. The relationship with age was limited and inconsistent; a greater preference in younger nursing students was found for working with older people [35, 36], but older students for working with people with dementia [28]. However, the majority found no relationship. Ethnicity or nationality was not commonly reported, but some associations were found [13, 31, 34, 37–39]. Religion was only explored in nursing students and the literature is inconsistent: three studies show a relationship [14, 39, 40], whilst two do not [41, 42].

#### Year of training

The research indicates that preferences are associated with earlier years in training in medical students [9, 43] and in nursing students [11, 36, 37, 44, 45]. However, this is made less clear by two contradictory studies in nursing students [10, 31].

#### Family characteristics

Preferences were associated with nursing students with parents with a positive attitude to older people, those who are not only children [46] and having a close relationship with an older adult [41, 46]. In medical students, no association with close relationships was found [13, 47] but those with higher intentions to work with older people reported a positive influence of friend or family member [32]. Qualitatively, one study in the USA suggested that students describe relationships with elders and perspectives on the role of the family in caring differently, depending on their preference for geriatrics [48].

#### Knowledge

In the quantitative literature, there was limited support for an association with knowledge. Only two nursing studies found a positive association [36, 49] while four did not [37, 39, 50, 51]. Qualitatively, five themes relating to knowledge were identified in nursing students [10, 35, 39, 45, 52]. These represented clinical skills and attributes of the students rather than objective knowledge and suggest that students may feel a personal deficit in their ability to work with older people that influences preferences. In medical students, one qualitative study found that a lack of knowledge about academic careers and experience with older people was the most cited barrier to pursuing geriatrics or an academic geriatric career [53].

#### Attitudes

Aside from the demographic variables, attitudes were the most researched construct in both nursing and medical studies, with strong evidence of a link between positive attitudes towards older people [28, 31, 35, 37, 39, 40, 42, 46, 49, 51, 54–59] and towards older patients [13, 33, 40, 41, 47, 60, 61].

### Course characteristics

There was no support in the quantitative literature for a relationship between various course characteristics and preferences such as type of geriatric course content [31, 62, 63], location [41] and types of course [13, 31, 49]. The only supporting factor was the type of nursing course; public vs private universities [31] and college or diploma vs university [42].

### Experiences of students

#### General experiences

In general, positive and negative experiences with older people, regardless of setting, were considered influential and regarded as a central theme for nursing [39, 58, 64, 65] and medical students [48, 53].

#### Previous experience

Experiences with older people before training in both medical and nursing students were explored repeatedly as a variable. There was only a single study of medical students that found an association [63], the majority did not [9, 13, 33, 47, 61]. For nursing students, evidence of an association is stronger. A significant relationship was demonstrated for previous experience [42, 46, 49, 64], paid work [30, 37, 58] and volunteering experience [41]. This was also supported qualitatively [52]. Yet equally, some studies found no association [31, 35, 62, 66]. Three studies suggested that the amount of experience is a factor [11, 30, 41].

#### Clinical placements

A relationship was found between positively rated placements and preferences in nursing [46, 67], and medical students [32]. In addition, there was a relationship between medical students who perceived that placements affected their preference choices, and their actual preferences [9]. Qualitative nursing studies have identified clinical placements as influencing preferences both positively and negatively [10, 64, 68, 69]. Quantitatively, a number of aspects of placements in nursing were associated including the pedagogical atmosphere, quality of supervisory relationship [66], the usefulness of feedback, supportiveness of nurse mentors and of care workers [67], and setting of placement, for example, care home versus general ward [31]. The role of mentors in placement was also supported qualitatively [52].

#### Educational interventions

In this systematic review, interventions were classed as an educational programme if they were purposely evaluated with a comparison group. There was some evidence that positive preferences were associated with taking part in these tailored programmes for medical [34, 59, 61, 70] and nursing students [40, 71, 72]. One study described how a longitudinal clerkship in dementia positively influenced medical students considering preferences related to dementia [70].

### Career characteristics

#### Professional development

This was a pertinent factor for nurses. Qualitatively it was suggested that student nurses viewed the potential for development in careers with older people negatively [11] and as having limited opportunities for progression [68]. Students reportedly sought new and different experiences post-qualification [45] and viewed older peoples’ services as an area to work later in their careers [68, 73]. Students with higher preference also rated that the potential opportunity to pursue a Clinical Nurse Specialist role (within older people’s settings) would be an influencing factor in their career decisions [42]. No support was found for medical students relating to concerns for professional development; in fact, one study noted that medical students mentioned the increased demand for geriatric care as a positive aspect of the career [53].

#### Financial and prestige considerations

Perceived limited financial rewards and professional status was found to affect preferences negatively, evidenced in quantitative variables [9, 67] and qualitative themes [28, 35, 53, 68, 74]. For example, being a geriatrician was seen as less glamorous [74] or ‘sexy’ [9] by medical students and nursing students perceived working with older people as having less professional status, with lower pay and respect [68].

#### Lifestyle considerations

One study investigated how lifestyle considerations may affect preferences for geriatric medicine; they found a non-significant association for general lifestyle considerations but found medical students who reported length of training as not being a barrier held higher preferences [9]. Another study found preferences for geriatric medicine higher in those who rated opportunities to travel as important when considering their career [34].

### Patient characteristics

#### Age of patient

Unsurprisingly there was an association found with preferences for working in fields related to older patients and; students wishing to work with older people [9, 67] and; students who did not feel that working with younger patients were more satisfying [9].

#### Communication difficulties

Difficulties communicating with patients was distinguished as a theme in qualitative studies of nurses [58, 73]. This was also found in medical students; one theme highlighted that some students reported that taking a medical history from older adults as being challenging [74]. Communication with patients with dementia was found as a central factor in a study exploring barriers to working in dementia care [28].

#### Nature of patients’ illness

The nature of patients’ illness, specifically chronicity and progression, was a recurring theme for nursing [39, 45, 75] and medical students [48, 53, 74, 76]. Nursing students cited patients’ lack of clinical recovery [45] and feeling of hopelessness in care [39, 75] as deterrents. For medical students, one study found working with older people was not popular because of the complexity of care, nature of health conditions, such as progression and that this was depressing [53]. This was also explored quantitatively in medical students; lower preferences were found in students who stated they would rather not work with chronically ill patients or perceived chronicity of patients as a barrier to pursuing geriatrics [9]. One study found that medical students described how older patients were often responsible for their health problems, and therefore treating them was less rewarding [48].

#### Disposition of patient and family

Two studies found student nurses described negative stereotypes of older adults when describing their lack of preference [11, 45]. One study found that student nurses cited conflicting views about working with older patients, including enjoying working with older people because they were independent, easy to communicate with and generally amenable, whereas other students reported that they didn’t want to work with older people because they were difficult and would complain [39]. Another theme from this study also found those that indicated they would prefer to work with the elderly demonstrated empathy with and understanding of the difficulties of ageing [39]. A unique issue within intentions to work with people with dementia was safety, as patients were described as potentially violent [28]. Medical findings indicated that students felt that older people may have unrealistic expectations for treatment outcomes [74] and may be frustrating to work with due to non-compliance [48]. Difficult family dynamics were explored as a factor for medical students but were not found to be associated [9].

### Work characteristics

#### ‘Boring and unchallenging’

This theme appears as a distinct factor in both medical and nursing students. Perception of clinical work with older people was discussed as boring and lacking challenge by nursing students [35, 45, 52, 58, 73, 75, 77] and medical students [48, 74]. In nursing, those who rated gerontological nursing as having diversity (in clinical practice) had a higher preference [42] and low preferences were related to a perception of exposure to limited variation in illness and experiences [28, 45].

#### Complexity

In medical students, one study identified the complexity of managing multiple problems in older people, with most seeing this as a negative aspect of the role, whereas others believed that it could bring opportunities for innovation [74]. Ethical issues [74] and the requirement to have a large generalist knowledge base [76] were also identified as difficulties. In terms of quantitative variables, one study found that the complexity of patients was not associated with preferences but students who agreed that a lack of comfort with ambiguity was a barrier to pursuing geriatrics, also held lower preferences [9].

#### Emotional nature of work

In nursing students, the work was described as emotionally challenging due to the nature of conditions [78]. This was also regarded as a central barrier to dementia care [28]. For medical students, caring for older people was seen as being emotionally draining and having an impact on psychological wellbeing, yet some felt that managing end of life care could be rewarding [74]. Nursing students cited fears, discomfort, and distress with death and ageing both in relation to witnessing patients dying and in confronting their own fears about death [45, 58]. One quantitative study found less anxiety with ageing being associated with higher preference in nursing students [46]. However, this was contrasted in one study of medical students; those more interested in geriatrics were more likely to discuss the fear of death or elderly, whereas those less interested described more emotional impact from other conditions; the authors suggested students may be drawn to the area they most fear [48].

#### Control and autonomy

This was a unique factor for nurses. Students described how they felt they had autonomy in the workplace, but feared this, due to a lack of appropriate organisational support [52, 68] and had responsibility yet did not have the agency to act and influence practice [52, 68]. This independence was also described as a positive attribute by some students [75]. Students were more motivated to work with older people if they could see a way to make a difference in their patients’ lives [65]. One quantitative study found that student nurses with higher preferences also rated nursing professionals as having greater professional powers in elderly care [42].

#### Environment

Nursing students with low preference describe the environment and work-life in negative terms [35, 65], including other staff being unskilled and unmotivated [75] and lack of financial resources affecting care [52]. Students with more positive preferences reported better working conditions [42, 75]. Medical students also described a lack of staff and a strained work environment as negatively affecting upon preferences [76].

#### The focus of quality of life as a barrier

Student perceptions around the impact they can have on patients and their role as a factor, especially if quality of life, rather than cure is the goal of treatment. One study noted the differences between those with interest in geriatrics and perceptions of the role of doctors; those who hold negative perceptions about geriatrics tending to highlight the frustration of not being able to ‘treat’ patients, whereas those interested in geriatrics focused on improving patient quality of life and consequent reward from this [48]. Medical students who were more interested in geriatrics were more likely to agree that the focus of patient quality of life (as opposed to cure) was not a barrier to pursuing geriatrics [9]. Nursing students recognised the need for person-centred care but felt they would not be able to work holistically and promote quality of life due to the realities of working practices, particularly for residents in long-term care [52, 65].

#### Heavy workload /physicality

The physically demanding nature of the work was a factor for nursing students in older people and dementia including manual handling and workload [28, 77].

#### Technical procedures

Higher preferences for geriatrics in medical students were associated with less importance of technical procedures [34] however; this was not related in another study [9].

#### Positives of work

For nursing students, a positive variable was the ability to provide continuity of care [67], and this was reflected qualitatively in the theme of ‘long term relationships’ [75]. For medical students, those interested described positives such as a slower pace with increased and longer-term patient contact [48]. Nursing students described work could be meaningful, enjoyable and rewarding [39, 75, 77]. One study described how nursing students felt that preferences would be encouraged by ‘developing a value for gerontology’ through developing relationships with patients in order to see them as individuals, and appreciating the complexity in aged care [78].

### Theory of planned behaviour

The theory of planned behaviour (TPB) was used as a theoretical model in four studies of nursing preferences; TPB is a model which seeks to explain influencing factors on behaviour [79]. It suggests that people’s behaviour is a rational outcome of considering their ability to perform the behaviour (perceived behavioural control), their beliefs in society and significant others opinions on the behaviour (subjective norms) and individual attitudes to the behaviour. In this reviewed literature, the ‘behaviour’ is a career working with older adults and ‘intention’ is the preference for working with older people. The majority of these studies looked at preferences as the primary outcome rather than behaviour. Only a single study looked at actual behaviour, which was associated with preferences [62]. There was support for attitudes (to behaviour), subjective norms and perceived behavioural control as factors associated with preferences [14, 31, 51, 62].

## Discussion

This review has outlined seven categories of potential factors contributing to the preferences of working with older people and provided a comprehensive overview of existing literature for medical and nursing students in this area. This model derived from the literature may have value in understanding healthcare students’ career preferences and designing education to promote work with older adults and people with dementia.

### Key findings and implications

#### The role of undergraduate education

Student preferences for working with older people appear to decrease during training. One explanation is that education has a role in shaping perceptions of the field as low status with an emphasis on technical specialities; this socialisation process is seen as a deterrent for aged care [11, 80]. This has been referred to as a ‘hidden curriculum’ [81]. Key impactful areas during education are clinical placements and educational interventions. The literature suggests these experiences can be key to forming preferences. Nevertheless, the quality of placements, not simply exposure, appears important for promoting professions related to older adults. Examples of quality nursing placement characteristics are found in descriptions of ‘enriched environments’ [64]. These are identified by delivering a sense of security and belonging for the students at the start of their placement, creating purpose and achievement through learning, and reinforcement of the value and significance of gerontology as a profession [64]. The contribution of mentors was also highlighted [52, 66, 67]. Clinical placements and educational interventions should be reviewed to assess the impact upon preferences towards older people. Knowledge on the mechanisms by which placements can influence preferences is limited, but they are suggested to influence via the factors outlined: attitudes; perception of the field; and student preparedness, knowledge and confidence [16, 64] However, robust evaluations of educational interventions in terms of preferences are lacking.

#### Perceived characteristics of work, patients and career

The characteristics of the work, patients and career, identified in the direct context of influencing preferences, provide insight into why students find working with older people unattractive. This includes the perception of the work as ‘boring’, emotionally challenging, the focus on patient quality of life as opposed to cure as a barrier, the nature of patients’ illness, and communication difficulties, as well as perceived negative aspects of older patients’ disposition. Together this indicates a perception that working with older people and dementia is less valued and challenging. The implication of this is that these perceived barriers may be reduced through education by: establishing the value and improving the profile of work with older people; including the importance and role of healthcare professionals in enhancing quality of life in chronic conditions; and by targeted skill development in perceived areas of difficulty, such as communication and emotional situations. However, while some of these perceptions can be challenged, we must acknowledge the reality of the environmental aspects and career limitations that students recognise. For example, inadequate older peoples services [2] and lack of prestige as described by doctors in geriatrics [82, 83]. Previous authors have suggested how these perceptions explain the unpopularity despite relative high attitudes to older people [31, 73]. Therefore, systemic changes are needed in older people’s services; however, this could be facilitated by inspiring newly qualified healthcare professionals who are able to drive these changes.

#### Preferences for working with people with dementia

There was a paucity of research in relation to dementia; only two studies explored preferences specifically in relation to working with people with dementia. Potential factors included: female gender; older students; characteristics of the work such as communication and emotional challenges; and educational interventions. Of the factors related to older people, those specifically of relevance include the value of work that appears to stem from the negative perception of chronic and progressive illness and the role of healthcare professionals facilitating quality of life rather than cure. This is pertinent to working with people with dementia.

Two studies mentioned dementia in relation to the importance of exposure to healthy adults to reduce stereotypical prejudices and promote working with older people [31, 78]. The question is how to reconcile this with the evident need for dementia education. A number of new educational programmes are being developed to meet this need [84, 85]. Results suggest the importance of positive clinical experiences and potential for educational interventions to influence preferences positively in dementia-related fields [70]. The implication of these results is that these interventions may offer a way to stimulate interest (both generalist and specialist) but robust evaluations are necessary.

#### Medical and nursing students

Similar factors were evidenced by both nursing and medical students, specifically, the perceptions of patients and characteristics of work as well as attitudes. The main divergence around was aspects of career pathways leading to differences. There was also more literature on nursing students with more diversity in the types of nursing preferences explored, this is likely due to the nursing career paths being relatively unstructured comparatively to medicine. The inclusion of both medical and nursing students is a strength of this review as this is a multi-professional issue and allows preferences to be view in this context of both wider policy and education.

### Future work

Significant gaps in research include an exploration of positive factors, longitudinal data, validated preferences measures and clear definitions of preferences. There is also a paucity of robust evaluations of education interventions and understanding of mechanisms of influence. The development of conceptual frameworks would be critical in helping to conceptualise these factors and the relationships between them. One clear area for future research is preferences related to dementia.

### Limitations

Studies were not excluded based on quality, which could have introduced bias into the review [25]. However, this was an exploratory review looking at possible factors with the aim to be comprehensive. Secondly, we have grouped different types of preferences together, although many studies did not define either what they meant by working with older adults or equate particular settings with working with older adults. Future work should provide definitions, including considering interpretations of student responders. Finally, the analysis was restricted to a narrative synthesis and therefore the magnitude of associations was not examined, giving no indication on the relative weighting of factors. Furthermore, we did not make a distinction between univariate links and multivariate analysis; given that many of the studies explored preferences not as the primary outcome, with multiple correlations being presented, there is the risk of type-1 error. However, this review has three main strengths: a rigorous systematic review methodology; it is novel in its inclusion of medical and nursing students in older adult’s preferences; and it is the first to explore preferences for working with people with dementia.

## Conclusion

Seven overall categories of factors were found and provide implications for education to promote working with older people. It was found that while there is a wide and varied literature relating to older adults, understanding of factors associated with working with dementia specifically is limited and is a key area for future research.

## Supplementary information

**Additional file 1.** Example Search. Example search terms: CINAHL 20/09/2019.

**Additional file 2.** Extraction Template. Data extraction form.

**Additional file 3.** Overview of Studies. Details of each included study.

**Additional file 4.** Summary of Factors. Overview of the factors resulting from the synthesis and supporting studies.

## Data Availability

The datasets supporting the conclusions of this article are included within the article and its additional files.

## Abbreviations

MMAT: Mixed Methods Appraisal Tool
TPB: Theory of planned behaviour
