# Supplementary material for: Preferences of nursing and medical students for working with older adults and people with dementia: a systematic review
Source: BMC Med Educ. 2020 Mar 30;20:92. doi: 10.1186/s12909-020-02000-z (PMC7106576; doi:10.1186/s12909-020-02000-z)
Supplement: Supplementary file 1 — Additional file 1. Example Search. Example search terms: CINAHL 20/09/2019. [file 12909_2020_2000_MOESM1_ESM.pdf]

MY EBSCOhost

Thursday, September 20, 2018 10:31:44 AM

#	Query	Limiters/Expanders	Last Run Via	Results
S17	S4 AND S11 AND S16	Search modes - Boolean/Phrase	Interface - EBSCOhost Research Databases<br>Search Screen - Advanced Search<br>Database - CINAHL Plus	43
S16	S12 OR S13 OR S14 OR S15	Search modes - Boolean/Phrase	Interface - EBSCOhost Research Databases<br>Search Screen - Advanced Search<br>Database - CINAHL Plus	28,435
S15	((student* ad j3 nurs*) or "medical student*" or "allied health* student*" or "health* student*")	Search modes - Boolean/Phrase	Interface - EBSCOhost Research Databases<br>Search Screen - Advanced Search<br>Database - CINAHL Plus	11,272
S14	healthcare students	Search modes - Boolean/Phrase	Interface - EBSCOhost Research Databases<br>Search Screen - Advanced Search<br>Database - CINAHL Plus	2,588
S13	"Medical Students"	Search modes - Boolean/Phrase	Interface - EBSCOhost Research Databases<br>Search Screen - Advanced Search<br>Database - CINAHL Plus	9,133
S12	"Nursing Students"	Search modes - Boolean/Phrase	Interface - EBSCOhost Research Databases<br>Search Screen - Advanced Search<br>Database - CINAHL Plus	15,877
S11	(S5 OR S6 OR S7 OR S8 OR S9 OR S10)	Search modes - Boolean/Phrase	Interface - EBSCOhost Research Databases<br>Search Screen - Advanced Search<br>Database - CINAHL Plus	819,235
S10	"older adult*" or "older people" or elder* or	Search modes - Boolean/Phrase	Interface - EBSCOhost Research Databases<br>Search Screen - Advanced	818,624

	dementia or geriatric* or aged		Search<br>Database - CINAHL Plus	
S9	MM "Dementia" OR MM "AIDS Dementia Complex" OR MM "Dementia with Lewy Bodies" OR MM "Presenile Dementia" OR MM "Semantic Dementia" OR MM "Senile Dementia" OR MM "Vascular Dementia"	Search modes - Boolean/Phrase	Interface - EBSCOhost Research Databases<br>Search Screen - Advanced Search<br>Database - CINAHL Plus	23,450
S8	"Elder Care"	Search modes - Boolean/Phrase	Interface - EBSCOhost Research Databases<br>Search Screen - Advanced Search<br>Database - CINAHL Plus	554
S7	"Geriatrics"	Search modes - Boolean/Phrase	Interface - EBSCOhost Research Databases<br>Search Screen - Advanced Search<br>Database - CINAHL Plus	8,145
S6	"Gerontology"	Search modes - Boolean/Phrase	Interface - EBSCOhost Research Databases<br>Search Screen - Advanced Search<br>Database - CINAHL Plus	2,028
S5	"Geriatric Patients"	Search modes - Boolean/Phrase	Interface - EBSCOhost Research Databases<br>Search Screen - Advanced Search<br>Database - CINAHL Plus	1,739
S4	S1 OR S2 OR S3	Search modes - Boolean/Phrase	Interface - EBSCOhost Research Databases<br>Search Screen - Advanced Search<br>Database - CINAHL Plus	8,076
S3	"career preference" or "career choice" or "intent* to work" or speciali*ation or "career intent*" or "special*ty choice" or "special*ty interest" or (Special* adj4 interest*)	Search modes - Boolean/Phrase	Interface - EBSCOhost Research Databases<br>Search Screen - Advanced Search<br>Database - CINAHL Plus	8,031
S2	"Occupational Choice"	Search modes -	Interface - EBSCOhost	48

		Boolean/Phrase	Research Databases<br>Search Screen - Advanced Search<br>Database - CINAHL Plus	
S1	"Occupational Preference"	Search modes - Boolean/Phrase	Interface - EBSCOhost Research Databases<br>Search Screen - Advanced Search<br>Database - CINAHL Plus	3
