# Supplementary material for: Preferences of nursing and medical students for working with older adults and people with dementia: a systematic review
Source: BMC Med Educ. 2020 Mar 30;20:92. doi: 10.1186/s12909-020-02000-z (PMC7106576; doi:10.1186/s12909-020-02000-z)
Supplement: Supplementary file 2 — Additional file 2. Extraction Template. Data extraction form. [file 12909_2020_2000_MOESM2_ESM.docx]

| BACKGROUND INFORMATION | |
| --- | --- |
| **Extracted by:** | |
| Reference: | |
| **STUDY DESIGN** | |
| Design |  |
| Country |  |
| Main Objectives |  |
| Student type |  |
| Response rate (Quant) |  |
| Number (Quant) |  |
| Number (Qual) |  |
| Data collection, Sampling  (for Qual) |  |
| Definition of career preference investigated | **Type of ‘preference’ and field of study**  **Examples include**;  Preference to work with older people  intent to specialise in geriatrics  Interest In working with people with dementia  attitude towards working with older patients  Include full definition by study if given. |
| Name of **career preference measure** **(Quant)** |  |
| Details of **career preference** Measurement used **(Quant)** | Give details of measurement used (e.g. Likert scale, rankings or application data).  Include the questions or choices of rankings used.  Source of measure (i.e. do they give a reference for it or was it developed for this study). |
| Results on relative popularity/ average preference **(Quant or Qual)** |  |
| Dementia Relevance | Include any mention of dementia in article (if article is not specifically looking at dementia career preferences). |
| Any other notes |  |
| MMAT Score | /5 |

**Quantitative data**

To include all factors explored in relation to career preferences only.

| **Name of variable (factor)** | **Details of Measurement**  **(of factor)** | **Sig** | **Association** | **Notes/univariate/multivariate stats** |
| --- | --- | --- | --- | --- |
| e.g AGE  e.g previous exp  e.g year of training  e.g country  (use name that is given by author) | Scale used, Reference | Sig.  or n.s  or Not reported  Sig must be set to at least >0.05  If the factor is explored by sub groups. BOLD.  For example:  **Male:** sig  **Female:** n.s | Only if Sig.  For all factors report in terms of its relationship from  low to high  Preference.  Positive  Negative  Descriptive: |  |
| **Details of intervention** | **Details of control/comparison** | **sig** | **Association** | **notes** |
| Components of intervention.  Aim of intervention | e.g pre/post, comparison groups, active controls | Sig.  or n.s  or Not reported  Sig must be set to at least <0.05  Include any sub groups. | Positive  Negative  Descriptive: |  |

**Qualitative data**

| **Qualitative Method details** | | |
| --- | --- | --- |
| Details: Include the type of data used, questions used and method of analysis.  In papers with different sections/ multiple aims (not just exploring career preferences), give an overview of all analysis but state if only a section is relevant to career preferences, and only extract those themes. | | |
| **Main Theme**  **(Factor)** | **Explanation** | **Quotes** |
|  |  |  |
