# Supplementary material for: Preferences of nursing and medical students for working with older adults and people with dementia: a systematic review
Source: BMC Med Educ. 2020 Mar 30;20:92. doi: 10.1186/s12909-020-02000-z (PMC7106576; doi:10.1186/s12909-020-02000-z)
Supplement: Supplementary file 3 — Additional file 3. Overview of Studies. Details of each included study. [file 12909_2020_2000_MOESM3_ESM.docx]

# Overview of Studies: Preferences’ of nursing and medical students for working with older adults and people with dementia: a systematic review.

| **Short Reference**  (Full references below) | | **Design/ Methods**  **MMAT Score**  **Out of ******* | **Participants**  Student type, Country Number:  Qt= Quantitative  Ql= Qualitative | **Main Objectives** |
| --- | --- | --- | --- | --- |
| 1 | **Abbey, J., et al. (2006)** | Qualitative (focus groups)  *** | Nursing, Australia  Qt: n/a  Ql: n=14 | Explores aspects of aging care placements in relation to students’ intentions to pursue a career in aged care. |
| 2 | **Alsenany, S. (2010)[A] Alsenany, S. and A. Al Saif (2012) [B][ full text not found]** | Mixed Methods (Thesis). Cross-sectional Surveys with open questions  (student data)  **** | Nursing, Saudi Arabia  Qt: Phase 1 n=566 Phase 2 n=200  Ql: Unknown | "The aims of this study are to explore the attitudes, knowledge, willingness, intentions and work preferences towards the care of older people among nursing students in the undergraduate nursing curricula in Saudi Arabia." |
| 3 | **Ayoǧlu, F. N., et al. (2014)** | Cross-sectional comparative survey  *** | Nursing and Medical, Turkey  Qt: n= 618 (Nurses n=339 Medics n=279)  Ql: n/a | "to evaluate the attitudes of nursing and medical students toward older people" |
| 4 | **Bagri, A. S. and R. Tiberius 5(2010).** | Qualitative (focus groups)  *** | Medical (allopathic),USA  Qt: n/a  Ql: n=30 | "ascertain medical students attitudes on Geriatrics" |
| 5 | **Ben Natan, M., et al. (2015).** | Cross-sectional survey  ***** | Nursing, Israel  Qt: n=200  Ql: n/a | To explore 'factors related to nursing students’ intention to work in geriatrics upon graduation |
| 6 | **Boyle, V., et al. (2014).** | Cross-sectional Survey  **** | Medical, New Zealand  Qt: n=711  Ql: n/a | "This study describes gender patterns of current specialty interest among medical students at the University of Auckland, and models the predictive effect of gender compared to other career influencing factors" |
| 7 | **Briscoe, V. J. (2004).** | Quasi-experimental study (Solomon four-group design, Survey pre/post intervention)  *** | Nursing, USA  Qt: n= 104 (Experimental n= 41, Control n=63)  Ql: n/a | To investigate attitudes and knowledge after a gerontology course, and the association of career preferences with attitude and knowledge. |
| 8 | **Brown, J., et al. (2008).** | Multi-method, multi-stage approach. Cross-sectional Survey and Qualitative Focus groups.  *** | Nursing, UK  Qt: n=718  Ql: not known | To identify the best practice for teaching care of older people, that supports positive interest in the field. |
| 9 | **Byszewski, A., et al. (2017).** | Quasi experimental study, pre/post survey after intervention  *** | Medical, Canada  Qt: n=93  Ql: n/a | Develop and evaluate an informational podcast on geriatrics |
| 10 | **Carlson, E. and E. Idvall (2015) [A]Carlson, E. (2015).[B]** | Cross-sectional survey (with open text)  *** | Nursing, Sweden  Qt: n=183 (1st yrs.) [A]  QL: n=224  (1st yrs. and 3rd yrs.) [B] | The aim of this study was to explore student nurses’ reasons for and against a future career in aged care" |
| 11 | **Che, C. C., et al. (2018)** | Cross-sectional survey  **** | Nursing, Malaysia  Qt: n= 1462  Ql: n/a | "determine the associations between demographical characteristics and working intentions towards older people, as well as to determine the predictors of intentions to work with older people among nursing students" |
| 12 | **Cheng, M., et al. (2015)** | Cross-sectional Survey  **** | Nursing, China  Qt: n=916  Ql: n/a | "The purpose of this study was to examine the motivation for choosing gerontological nursing as a career and to identify the associated factors among student nurse" |
| 13 | **Chi, M. J., et al. (2016).** | Cross-sectional survey  ***** | Nursing, Taiwan  Qt: n=612  Ql: n/a | "This study aimed to explore Taiwanese nursing students’ willingness to work with older persons and factors associated with this" |
| 14 | **Chua, M. P., et al. (2008).** | Cross-sectional survey  **** | Medical, Singapore  Qt: n=244  Ql: n/a | "This study determined medical students’ attitudes towards older people and their willingness to consider a career in Geriatric Medicine." |
| 15 | **Curran, M. A., et al. (2015)** | Cross-sectional survey (with open questions.)  ** | Medical, USA  Qt: n=27  Ql: n=27 | "This study sought to obtain medical students’ perspectives on barriers and facilitators toward pursuing a career in academics and/or in geriatric psychiatry or geriatric medicine." |
| 16 | **Darling, R., et al. (2017)** | Cross-sectional survey  *** | Nursing, Turkey  Qt: 468  Ql: n/a | "The purposes of this study were to assess the overall attitudes of nursing students at a Turkish university toward the elderly and to identify the effects of demographic and social factors on attitudes" |
| 17 | **de Guzman, A. B., et al. (2013)** | Cross-sectional survey  **** | Nursing, Philippines  Qt: n=839  Ql: n/a | "Anchored on the key constructs of Ajzen’s Theory of Planned Behaviour (1985), this paper seeks to test a model that explores the influence of knowledge, attitude, and caring behaviour on nursing students’ behavioural intention toward geriatric care." |
| 18 | **Diachun, L. L., et al. (2006)** | Longitudinal Survey (1st and 2nd yr.) with open text questions  **** | Medical, Canada  Qt: n= 108 matched (T1 n= 121,  T2 n= 118)  Ql: n/a | "This study explored student interest in and barriers and enticements to geriatric medicine as a career choice." |
| 19 | **Diachun, L. L., et al. (2006).** | Quasi-experimental design (longitudinal survey initially after intervention, and one year later)  ** | Medical, Canada  Qt: n=42  Ql: n/a | “This study tested the hypothesis that experiential education is superior to a traditionally didactic approach by evaluating whether superior knowledge scores measures after a participatory, experiential undergraduate geriatrics learning session were maintained after 1 year. Students’ attitudes toward older people and interest in geriatric medicine were also evaluated 1 year after undergoing a didactic or participatory first-year learning session in geriatrics” |
| 20 | **Duggan, S., et al. (2013).** | Qualitative (focus groups)  ***** | Nursing, UK (Ireland)  Qt: n/a  Ql: n=32 | "To explore students’ perceptions of working with older people and the extent to which their preregistration curriculum is preparing them for this role." |
| 21 | **Dunkle, S. E. and R. S. Hyde (1995)** | Cross-sectional survey with longitudinal component  ** | Nursing, USA  Qt: n=85  Ql: n/a | "Identify factors that influence physical therapist and nursing RNS students intentions and behaviours towards working with the elderly, and to test the application of the Theory of Reasoned Action." |
| 22 | **Fagerberg, I., et al. (2000).** | Longitudinal Qualitative study (interviews and Diaries)  ***** | Nursing, Sweden  Qt: n/a  Ql: n=27 | To understand the reasons for where student nurses would like to work after graduation, in relation to elderly care. |
| 23 | **Fitzgerald, J. T., et al. (2003).** | cross-sectional survey  **** | Medical, USA  Qt: n=171  Ql: n/a | This study examined medical student’s interest in geriatrics “Are knowledge, positive attitudes, and prior experience with older adults associated with an interest in geriatric medicine?" |
| 24 | **Fox, S. D. and J. E. Wold (1996)** | Quasi-Experimental (pre/post survey with open questions- [not extracted])  *** | Nursing, USA  Qt: n=144  Ql: n/a | "evaluate perceived learning and attitude changes following a gerontological placement in final year nursing students" |
| 25 | **Gates, K., et al. (2009).** | Qualitative (Focus Groups)  *** | Nursing, Canada  Qt: n/a  Ql: n=27 | To understand student “perspectives, experiences, values, ideas and opinions” about working with older adults. |
| 26 | **Gonzales, E., et al. (2010)** | Quasi-experimental (with pre/post survey with control and focus groups)  *** | Medical, USA  Qt: n= 208  (For preference data only: T1: Treatment n =104,Comparison n= 92.T2:Treatment n = 87 Comparison n= 76)  Ql: n/a | To explore the impact of ‘Vital Visionaries’ (an intergeneration art program) on attitudes toward older adults, perception of commonality with older adults, and career plans. |
| 27 | **Gould, O. N., et al. (2012).** | Longitudinal Survey (beginning and end of year, for 1st, 2nd and 4th years) with open questions.  *** | Nursing, Canada  Qt: n= 58 matched (T1 n=170, T2 n=75)  Ql: n=195 | "This study investigates novice(first and second years) and experienced ( fourth years)student nurses’ attitudes about caring for patients across the lifespan" |
| 28 | **Happell, B. (1999) [A] Happell, B. and J. Brooker (2001) [B] Happell, B. (2002).[C] Happell, B. (2002). [D]** | Longitudinal Questionnaire (with open questions)  *** | Nursing, Australia  Qt: T1 n= 793 T2 n=521  Ql: n/a | To explore the career preferences of nursing students at the beginning and end of their course. |
| 29 | **Haron, Y., et al. (2013)** | Cross-sectional survey  **** | Nursing, Israel  Qt: n=486  Ql: n/a | "To gather last-year student nurses’ views on geriatric nursing as a career choice and identify the factors behind those views.” |
| 30 | **Henderson, J., et al. (2008)** | Cross-sectional Survey (with open questions)  **** | Nursing, Australia  Qt: n=262  Ql: n/a | To assess attitudes in first-year nursing students to older people and working with older people. |
| 31 | **Herdman, E. (2002).** | Qualitative (Semi-structured questionnaires)  **** | Nursing, Hong Kong  Qt: n=96  Ql: n/a | "to explore student nurses’ reasons for undertaking a nursing degree course, their career specialty preferences, the rationale behind these preferences and their views of working with elderly patients." |
| 32 | **Hughes, N. J., et al. (2008).** | 1)Cross-sectional survey (with 1st year students) 2)Quasi-experimental, Pre/Post intervention Survey (with 4th year students)  ** | Medical, UK (Scotland)  Qt: 1) n= 163  2) n= 70  matched  Ql: n/a | "To evaluate the attitudes of first- and fourth year medical students toward older people and the relationship between these attitudes and possible career choice." |
| 33 | **Hweidi, I. M. and S. M. Al-Obeisat (2006)** | Cross-sectional survey  **** | Nursing, Jordan  Qt: n=243  Ql: n/a | "The purpose of this study was to identify Jordanian nursing students’ attitudes towards older people and to consider whether the attitudes of the selected sample had any bearing on the care provided for this client group" |
| 34 | **Jefferson, A. L., et al. (2012).** | Quasi-experimental  (pre/post Survey following intervention , with reflective essays)  ***** | Medical, USA  Qt: n=45  Ql: n=45 | "The purpose of this study is to evaluate the PAIRS Program and its effectiveness in enhancing medical education as a service-learning activity and replication model for the Buddy Program^TM^." |
| 35 | **King, B. J., et al. (2013).** | Longitudinal mixed-methods (Survey at y1, y2, y3 and y4, and focus groups)  *** | Nursing, USA  Qt: n= 80 (T1 n= 80 T2 n= 43 T3 n=75 T4 = 66)  Ql: n=10 | "The purpose of this longitudinal mixed methods study is to describe and explain student nurse attitudes and preference changes over time" |
| 36 | **Kloster, T., et al. (2007).** | Longitudinal Survey (start and end of course) with open text questions  *** | Nursing, Norway  Qt: n/a  Ql: n=187 [for content analysis for reasons wanting to/ not wanting to work in 'aged care institution') | "identify Norwegian undergraduate nursing students’ career preferences at the beginning and end of their nursing education programme, together with their reasons for these preferences” |
| 37 | **Koehler, A. R., et al. (2016** | Quasi-experimental (pre- and post-test survey following intervention)  *** | Nursing, USA  Qt: n= 266 matched  (T1: 266 T2: 266)  Ql: n/a | "To examine the impact of a stand-alone course in gerontological nursing on undergraduate nursing students' perceptions of working with older adults and career intentions." |
| 38 | **Koskinen, S. (2016) [A] Koskinen, S., et al. (2012) [B]** | Multi phased design (Thesis) 1) Cross-sectional Survey 2) Quasi-experimental pre-post-test design evaluation  **** | Nursing, Finland  Qt: 1) N= 183  2) N=87  (intervention n=40 , teaching as usual n=36)  Ql: n/a | "The purpose of this two-phased study is to examine the interest of nursing students in choosing a career in older people nursing. First, the scoping phase explores the different premises for choosing older people nursing as a career. Second, the evaluation phase investigates the outcomes of the developed educational intervention involving older people as promoters of choosing a career in older people nursing, factors related to these outcomes, and experiences with educational intervention. The ultimate goal is to encourage more nursing students to choose older people nursing as their career." |
| 39 | **Lea, E., et al. (2016).** | Quasi-experimental design  (pre/post Survey following intervention)  *** | Nursing, Australia  Qt: n=71  (T2. Post Survey data explored only)  Ql: n/a | "To investigate which aspects of student nurses’ experiences of residential aged care facility clinical placements affect perceived likelihood of choosing a career in residential aged care post graduation." |
| 40 | **Lee, A. C. K., et al. (2006)** | Cross-sectional survey  *** | Nursing, Hong Kong  Qt: n=219  Ql: n/a | "The present study aimed to examine the knowledge and working preferences toward the elderly among undergraduate nursing students in Hong Kong. " |
| 41 | **Lu, W.-H., et al. (2010).** | Quasi-experimental (pre/ post survey with focus group)  *** | Medical, USA  Qt: n=137  (Intervention n=46 Control n=91)  Ql: n=9 (intervention) | "The purpose of this study was to examine the impact of an extracurricular geriatric program on medical students’ knowledge of, and attitudes toward, the elderly and their interest in studying geriatric medicine" |
| 42 | **McKenzie, E. L. and P. M. Brown (2014).** | Cross-sectional survey (with open text)  *** | Nursing, Australia  Qt: n=135  Ql: n=116 | “This study aimed to investigate factors influencing nursing students’ intentions to work in dementia care and to explore perceived barriers to working in dementia care” |
| 43 | **Ni Chroinin, D., et al. (2013).** | Cross-sectional survey  ** | Medical, Republic of Ireland  Qt: n=274  Ql: n/a | "(i) career choices of senior medical students; (ii) interest in geriatric medicine; (iii) factors influencing such choices; and (iv) the impact of a 6-week Medicine in the Community module." |
| 44 | **Pan, I. J., et al. (2009)** | Cross-sectional survey  **** | Nursing, Taiwan  Qt: n=362  Ql: n/a | "The aim of this research was to establish Taiwanese undergraduate nursing students 'attitudes toward older people. " |
| 45 | **Rathnayake, S., et al. (2016)** | Cross-sectional survey  **** | Nursing, Sri-Lanka  Qt: n=98  Ql: n/a | "Examine the attitudes of undergraduate nursing students toward older people and their willingness to work with older people in Sri Lanka." |
| 46 | **Robbins, T. D., et al. (2011).** | Cross-sectional Survey  **** | Medical, UK  Qt: n=1,562  Ql: n/a | "To describe attitudes to, and perceptions of, geriatric medicine among the students of all UK medical schools." |
| 47 | **Samra, R. (2013).** | Qualitative (individual interviews)  [Sub study 3 in Thesis]  ***** | Medical, UK  Qt: n/a  Ql: n=22 | To conceptualise medical student and doctors attitudes towards older people (in UK hospital settings). The aim of Sub study 3 was to "explore factors involved in junior doctors’ decisions to pursue or not pursue geriatric medicine as a career choice" |
| 48 | **Schigelone, A. S. and B. Ingersoll-Dayton (2004).** | Qualitative study (Individual interviews)  **** | Medical, USA  Qt: n/a  Ql: n=20 | To explore the reasons for first-year medical students interest or uninterested in pursuing geriatrics. |
| 49 | **Shen, J. and L. D. Xiao (2012).** | Cross-sectional survey (with open questions)  **** | Nursing, China  Qt: n=622  Ql: n/a | To explore factors affecting nursing students' intention to work with older people. |
| 50 | **Stevens, J. A. (2011).** | Longitudinal Survey  (3 time points, start , middle, end of course)  *** | Nursing, Australia  Qt: n= 150 Matched data (T1 n= 203, T2 n=189, T3 n= 160)  Ql: n/a | “The main research objectives in this study were to (1) develop a proﬁle of nursing career preferences and the rationale underpinning those choices and (2) compare these results with other literature to indicate if this proﬁle is changing.” |
| 51 | **Swanlund, S. and A. Kujath (2012).** | Cross-sectional study (with open questions)  ** | Nursing, USA  Qt: n=50  Ql: n/a | "To examine nursing students’ attitudes toward older adults.. at all level of nursing education.." four research questions including ‘what factors would help increase student interest in gerontology?’ |
| 52 | **Voogt, S. J., et al. (2008)** | Cross-sectional survey  **** | Medical, USA  Qt: n=231  Ql: n/a | "the purpose of this study was to more clearly understand the specific factors involved in choosing geriatric medicine as a career path among first-year medical students." |
| 53 | **Xiao, L. D., et al. (2013)** | Cross-sectional comparative survey  **** | Nursing, Australia and China  Qt: n= 466  (Aus. n= 262, China n= 204)  Ql; 460 | “The aim of this study was to compare Australian and Chinese nursing students’ attitudes and intentions to care for the elderly and the factors affecting these intentions.” |
| 54 | **Zakari, N. M. A. (2005)** | Cross-sectional Survey (With open text questions  ***** | Nursing, Saudi Arabia  QT: n=506  Ql: n=33 | Examine the relationships between attitudes, knowledge, willingness and intention to work with the elderly and predictors of intention. |
| 55 | **Zhang, S., et al. (2016).** | Cross-sectional survey  **** | Nursing, China  Qt: n=382  Ql: n/a | "The purpose of this study is to explore the relationships among knowledge about aging, care willingness, attitude towards the elderly and gratitude." |
| 56 | **Zisberg, A. P. R. N., et al. (2015)** | Cross-sectional survey  **** | Nursing, Israel  Qt: n=224  Ql: n/a | "The aim of this study was to examine the effect of nursing education on students’ knowledge, attitudes, and preferences to work with older adults in an ethnically diverse Israeli society" |

References

| 1 | Abbey, J., Abbey, B., Bridges, P., Elder, R., Lemcke, P., Liddle, J., & Thornton, R. (2006). Clinical placements in residential aged care facilities: the impact on nursing students' perception of aged care and the effect on career plans. Australian Journal of Advanced Nursing, 23(4), 14-19. |
| --- | --- |
| 2A | Alsenany, S. (2010). An exploration of the attitudes, knowledge, willingness and future intentions to work with older people among Saudi nursing students in baccalaureate nursing schools in Saudi Arabia. |
| 2B | Alsenany, S., & Al Saif, A. (2012). Comparison between Saudi and British nursing students toward working with older people. Journal of American Science, 8(7), 316-328. |
| 3 | Ayoǧlu, F. N., Kulakçı, H., Ayyıldız, T. K., Aslan, G. K., & Veren, F. (2014). Attitudes of Turkish Nursing and Medical Students Toward Elderly People. Journal of Transcultural Nursing, 25(3), 241-248. doi:10.1177/1043659613515527 |
| 4 | Bagri, A. S., & Tiberius, R. (2010). Medical student perspectives on geriatrics and geriatric education. Journal of the American Geriatrics Society, 58(10), 1994-1999. doi:10.1111/j.1532-5415.2010.03074.x |
| 5 | Natan, B. M., Danino, S., Freundlich, N., Barda, A., & Mor Yosef, R. (2015). Intention of Nursing Students to Work in Geriatrics. Research in Gerontological Nursing, 8(3), 140-147. doi:10.3928/19404921 -20150219-03 |
| 6 | Boyle, V., Shulruf, B., & Poole, P. (2014). Influence of gender and other factors on medical student specialty interest. New Zealand Medical Journal, 127(1402), 78-87. |
| 7 | Briscoe, V. J. (2004). The effects of gerontology nursing teaching methods on nursing student knowledge, attitudes, and desire to work with older adult clients. Ph.D., 127 p-127 p. |
| 8 | Brown, J., Nolan, M., Davies, S., Nolan, J., & Keady, J. (2008). Transforming students' views of gerontological nursing: Realising the potential of 'enriched' environments of learning and care: A multi-method longitudinal study. International journal of nursing studies, 45(8), 1214-1232. doi:10.1016/j.ijnurstu.2007.07.002 |
| 9 | Byszewski, A., Bezzina, K., & Latrous, M. (2017). What Kind of Doctor Do You Want to Be? Geriatric Medicine Podcast as a Career Planning Resource. BioMed Research International, 1-6. doi:10.1155/2017/6183148 |
| 10A | Carlson, E. (2015). Meaningful and enjoyable or boring and depressing? The reasons student nurses give for and against a career in aged care. Journal of Clinical Nursing, 24(3-4), 602-604. doi:10.1111/jocn.12425 |
| 10B | Carlson, E., & Idvall, E. (2015). Who wants to work with older people? Swedish student nurses' willingness to work in elderly care--a questionnaire study. Nurse education today, 35(7), 849-853. doi:https://dx.doi.org/10.1016/j.nedt.2015.03.002 |
| 11 | Che, C. C., Chong, M. C., & Hairi, N. N. (2018). What influences student nurses' intention to work with older people? A cross-sectional study. International journal of nursing studies, 85, 61-67. doi:https://dx.doi.org/10.1016/j.ijnurstu.2018.05.007 |
| 12 | Cheng, M., Cheng, C., Tian, Y., & Fan, X. (2015). Student nurses' motivation to choose gerontological nursing as a career in China: a survey study. Nurse education today, 35(7), 843-848. doi:https://dx.doi.org/10.1016/j.nedt.2015.03.001 |
| 13 | Chi, M. J., Shyu, M. L., Wang, S. Y., Chuang, H. C., & Chuang, Y. H. (2016). Nursing students’ willingness to care for older adults in Taiwan. Journal of Nursing Scholarship, 48(2), 172-178. |
| 14 | Chua, M. P., Tan, C. H., Merchant, R., & Soiza, R. L. (2008). Attitudes of first-year medical students in Singapore towards older people and willingness to consider a career in geriatric medicine. Annals of the Academy of Medicine, Singapore, 37(11), 947-951. |
| 15 | Curran, M. A., Black, M., Depp, C. A., Iglewicz, A., Reichstadt, J., Palinkas, L., & Jeste, D. V. (2015). Perceived barriers and facilitators for an academic career in geriatrics: Medical students’ perspectives. Academic Psychiatry, 39(3), 253-258. doi:10.1007/s40596-014-0208-6 |
| 16 | Darling, R., Sendir, M., Atav, S., & Buyukyilmaz, F. (2017). Undergraduate nursing students and the elderly: An assessment of attitudes in a Turkish university. Gerontology & Geriatrics Education, 1-12. doi:https://dx.doi.org/10.1080/02701960.2017.1311883 |
| 17 | de Guzman, A. B., Jimenez, B. C. B., Jocson, K. P., Junio, A. R., Junio, D. E., Jurado, J. B. N., & Justiniano, A. B. F. (2013). Filipino Nursing Students' Behavioral Intentions toward Geriatric Care: A Structural Equation Model (SEM). Educational Gerontology, 39(3), 138-154. |
| 18 | Diachun, L. L., Hillier, L. M., & Stolee, P. (2006). Interest in geriatric medicine in Canada: how can we secure a next generation of geriatricians? Journal of the American Geriatrics Society, 54(3), 512-519. doi:10.1111/j.1532-5415.2005.00610.x |
| 19 | Diachun, L. L., Dumbrell, A. C., Byrne, K., & Esbaugh, J. (2006). But Does It Stick? Evaluating the Durability of Improved Knowledge Following an Undergraduate Experiential Geriatrics Learning Session. Journal of the American Geriatrics Society, 54(4), 696-701. doi:10.1111/j.1532-5415.2006.00656.x |
| 20 | Duggan, S., Mitchell, E. A., & Moore, K. D. (2013). 'With a bit of tweaking...we could be great'. An exploratory study of the perceptions of students on working with older people in a preregistration BSc (Hons) Nursing course. International Journal of Older People Nursing, 8(3), 207-215. doi:https://dx.doi.org/10.1111/j.1748-3743.2012.00317.x |
| 21 | Dunkle, S. E., & Hyde, R. S. (1995). Predictors and subsequent decisions of physical therapy and nursing students to work with geriatric clients: an application of the Theory of Reasoned Action. Physical Therapy, 75(7), 614-620. |
| 22 | Fagerberg, I., Winblad, B., & Ekman, S. L. (2000). Influencing aspects in nursing education on Swedish nursing students' choices of first work area as graduated nurses. Journal of Nursing Education, 39(5), 211-218. |
| 23 | Fitzgerald, J. T., Wray, L. A., Halter, J. B., Williams, B. C., & Supiano, M. A. (2003). Relating Medical Students' Knowledge, Attitudes, and Experience to an Interest in Geriatric Medicine. The Gerontologist, 43(6), 849-855. doi:10.1093/geront/43.6.849 |
| 24 | Fox, S. D., & Wold, J. E. (1996). Baccalaureate student gerontological nursing experiences: raising consciousness levels and affecting attitudes. Journal of Nursing Education, 35(8), 348-355. |
| 25 | Gates, K., Santos, E. J., Nguyen, M., Granovskaya, I., Servidio, A., & Turzanski, M. (2009). Gerontology education initiatives in the health sciences: seeking advice from students in focus group conversations. Perspectives, 33(3), 6-13. |
| 26 | Gonzales, E., Morrow-Howell, N., & Gilbert, P. (2010). Changing medical students' attitudes toward older adults. Gerontology & Geriatrics Education, 31(3), 220-234. doi:10.1080/02701960.2010.503128 |
| 27 | Gould, O. N., MacLennan, A., & Dupuis-Blanchard, S. (2012). Career preferences of nursing students. Canadian Journal on Aging, 31(4), 471-482. doi:10.1017/S0714980812000359 |
| 28A | Happell, B. (1999). When I grow up I want to be a...? Where undergraduate student nurses want to work after graduation. Journal of advanced nursing, 29(2), 499-505. |
| 28B | Happell, B. (2002a). Nursing home employment for nursing students: valuable experience or a harsh deterrent? Journal of advanced nursing, 39(6), 529-536. |
| 28C | Happell, B. (2002b). The role of nursing education in the perpetuation of inequality. Nurse education today, 22(8), 632-640. |
| 28D | Happell, B., & Brooker, J. (2001). Who will look after my grandmother? Attitudes of student nurses toward the care of older adults. Journal of gerontological nursing, 27(12), 12-17. |
| 29 | Haron, Y., Levy, S., Albagli, M., Rotstein, R., & Riba, S. (2013). Why do nursing students not want to work in geriatric care? A national questionnaire survey. International journal of nursing studies, 50(11), 1558-1565. doi:10.1016/j.ijnurstu.2013.03.012 |
| 30 | Henderson, J., Xiao, L., Siegloff, L., Kelton, M., & Paterson, J. (2008). 'Older people have lived their lives': First year nursing students' attitudes towards older people. Contemporary Nurse, 30(1), 32-45. doi:10.5172/conu.673.30.1.32 |
| 31 | Herdman, E. (2002). Challenging the discourses of nursing ageism. International journal of nursing studies, 39(1), 105-114. |
| 32 | Hughes, N. J., Soiza, R. L., Chua, M., Hoyle, G. E., MacDonald, A., Primrose, W. R., & Seymour, D. G. (2008). Medical student attitudes toward older people and willingness to consider a career in geriatric medicine. Journal of the American Geriatrics Society, 56(2), 334-338. doi:10.1111/j.1532-5415.2007.01552.x |
| 33 | Hweidi, I. M., & Al-Obeisat, S. M. (2006). Jordanian nursing students' attitudes toward the elderly. Nurse education today, 26(1), 23-30. |
| 34 | Jefferson, A. L., Cantwell, N. G., Byerly, L. K., & Morhardt, D. (2012). Medical student education program in Alzheimer's disease: the PAIRS Program. BMC medical education, 12, 80. doi:https://dx.doi.org/10.1186/1472-6920-12-80 |
| 35 | King, B. J., Roberts, T. J., & Bowers, B. J. (2013). Nursing student attitudes toward and preferences for working with older adults. Gerontology & Geriatrics Education, 34(3), 272-291. doi:10.1080/02701960.2012.718012 |
| 36 | Kloster, T., Høie, M., & Skår, R. (2007). Nursing students' career preferences: A Norwegian study. Journal of advanced nursing, 59(2), 155-162. doi:10.1111/j.1365-2648.2007.04276.x |
| 37 | Koehler, A. R., Davies, S., Smith, L. R., Hooks, T., Schanke, H., Loeffler, A., . . . Ratzlaff, N. (2016). Impact of a stand-alone course in gerontological nursing on undergraduate nursing students' perceptions of working with older adults: A Quasi-experimental study. Nurse education today, 46, 17-23. doi:10.1016/j.nedt.2016.06.015 |
| 38A | Koskinen, S. (2016). Nursing students and older people nursing. Towards a future career. |
| 38B | Koskinen, S., Hupli, M., Katajisto, J., & Salminen, L. (2012). Graduating Finnish nurse students' interest in gerontological nursing--a survey study. Nurse education today, 32(4), 356-360. doi:https://dx.doi.org/10.1016/j.nedt.2011.05.015 |
| 39 | Lea, E., Mason, R., Eccleston, C., & Robinson, A. (2016). Aspects of nursing student placements associated with perceived likelihood of working in residential aged care. Journal of Clinical Nursing, 25(5-6), 715-724. doi:10.1111/jocn.13018 |
| 40 | Lee, A. C. K., Wong, A. K. P., & Loh, E. K. Y. (2006). Score in the Palmore’s Aging Quiz, knowledge of community resources and working preferences of undergraduate nursing students toward the elderly in Hong Kong. Nurse education today, 26(4), 269-276. |
| 41 | Lu, W.-H., Hoffman, K. G., Hosokawa, M. C., Gray, M. P., & Zweig, S. C. (2010). First year medical students' knowledge, attitudes, and interest in geriatric medicine. Educational Gerontology, 36(8), 687-701. |
| 42 | McKenzie, E. L., & Brown, P. M. (2014). Nursing students' intentions to work in dementia care: Influence of age, ageism, and perceived barriers. Educational Gerontology, 40(8), 618-633. doi:10.1080/03601277.2013.863545 |
| 43 | Ni Chroinin, D., Cronin, E., Cullen, W., O'Shea, D., Steele, M., Bury, G., & Kyne, L. (2013). Would you be a geriatrician? Student career preferences and attitudes to a career in geriatric medicine. Age & Ageing, 42(5), 654-657. doi:https://dx.doi.org/10.1093/ageing/aft093 |
| 44 | Pan, I. J., Edwards, H., & Chang, A. (2009). Taiwanese Nursing Students' Attitudes Toward Older People. Journal of gerontological nursing, 1-6. doi:https://dx.doi.org/10.3928/00989134-20090903-01 |
| 45 | Rathnayake, S., Athukorala, Y., & Siop, S. (2016). Attitudes toward and willingness to work with older people among undergraduate nursing students in a public university in Sri Lanka: A cross sectional study. Nurse Educ Today, 36, 439-444. doi:10.1016/j.nedt.2015.10.007 |
| 46 | Robbins, T. D., Crocker-Buque, T., Forrester-Paton, C., Cantlay, A., Gladman, J. R. F., & Gordon, A. L. (2011). Geriatrics is rewarding but lacks earning potential and prestige: responses from the national medical student survey of attitudes to and perceptions of geriatric medicine. Age & Ageing, 40(3), 405-408. doi:ageing/afr033 |
| 47 | Samra, R. (2013). Medical students; and doctors attitudes toward older patients and their care: what do we known and where do we go from here? , Ph.D. |
| 48 | Schigelone, A. S., & Ingersoll-Dayton, B. (2004). Some of my Best Friends are Old: A Qualitative Exploration of Medical Students' Interest in Geriatrics. Educational Gerontology, 30(8), 643-661. doi:10.1080/03601270490483887 |
| 49 | Shen, J., & Xiao, L. D. (2012). Factors affecting nursing students' intention to work with older people in China. Nurse education today, 32(3), 219-223. doi:10.1016/j.nedt.2011.03.016 |
| 50 | Stevens, J. A. (2011). Student nurses’ career preferences for working with older people: A replicated longitudinal survey. International journal of nursing studies, 48(8), 944-951. doi:10.1016/j.ijnurstu.2011.01.004 |
| 51 | Swanlund, S., & Kujath, A. (2012). Attitudes of baccalaureate nursing students toward older adults: a pilot study. Nursing Education Perspectives, 33(3), 181-183. |
| 52 | Voogt, S. J., Mickus, M., Santiago, O., & Herman, S. E. (2008). Attitudes, experiences, and interest in geriatrics of first-year allopathic and osteopathic medical students. Journal of the American Geriatrics Society, 56(2), 339-344. doi:10.1111/j.1532-5415.2007.01541.x |
| 53 | Xiao, L. D., Shen, J., & Paterson, J. (2013). Cross-cultural comparison of attitudes and preferences for care of the elderly among Australian and Chinese nursing students. Journal of Transcultural Nursing, 24(4), 408-416. doi:10.1177/1043659613493329 |
| 54 | Zakari, N. M. A. (2005). Attitudes toward the elderly and knowledge of aging as correlates to the willingness and intention to work with elderly among Saudi nursing students. Ph.D., 236 p-236 p. |
| 55 | Zhang, S., Liu, Y.-h., Zhang, H.-f., Meng, L.-n., & Liu, P.-x. (2016). Determinants of undergraduate nursing students' care willingness towards the elderly in China: Attitudes, gratitude and knowledge. Nurse education today, 43, 28-33. |
| 56 | Zisberg, A. P. R. N., Topaz, M. M. A. R. N., & Band-Wintershtein, T. P. (2015). Cultural- and Educational-Level Differences in Students Knowledge, Attitudes, and Preferences for Working With Older Adults: An Israeli Perspective. Journal of Transcultural Nursing, 26(2), 193. |
