# Supplementary material for: Preferences of nursing and medical students for working with older adults and people with dementia: a systematic review
Source: BMC Med Educ. 2020 Mar 30;20:92. doi: 10.1186/s12909-020-02000-z (PMC7106576; doi:10.1186/s12909-020-02000-z)
Supplement: Supplementary file 4 — Additional file 4. Summary of Factors. Overview of the factors resulting from the synthesis and supporting studies. [file 12909_2020_2000_MOESM4_ESM.docx]

|  | Nursing students | Medical students | Nursing students | Medical students |  |
| --- | --- | --- | --- | --- | --- |
| **Factor** | **Variables (Quant)**  Number= Reference (See key below)  Significant finding= Sig (P<0.05) OR Non-significant = n.s  Association with preferences (recorded only if sig):  Positive= (+) , negative (-) or descriptive (Vs.) | | **Themes (Qual)**  Number= Reference (See key below) | | Summary of association with preferences |
| **CATEGORY 1: STUDENT CHARACTERISTICS** | | | | | |
| **Age** | 56: n.s, 13: n.s, 29: n.s, 10: n.s,  11: n.s, 21: n.s, 30: n.s.  49: Sig (-), 40: Sig (-). 42: Sig(+) | 14: n.s, 32: n.s, 52: n.s, 18: n.s. |  |  | Conflicting evidence on age. For nurses, Younger for older people, older for dementia. |
| **Gender** | 38: Sig (female), 11: Sig (Female)  5: Gender Sig (Male).  56: n.s, 12: n.s, 13: n.s, 29: n.s, 30: n.s, 10: n.s. | 6: Sig (Female), 14: Sig (Female), 43: Sig (Female), 18: Sig (female)  23: n.s, 32 n.s, 52: gender. n.s. |  |  | Evidence of link with gender (female), stronger in medical students. |
| **Year of training** | 56: Sig (-), 27: Sig(-) ,40: Sig(-), 50: Sig(-), 28: Sig(-), 11: Sig (3 yr. higher Vs. 1-4), 35: Sig(+)  49: n.s, 54: n.s. | 18: Sig(-), 19: Sig (-) |  |  | Negative association  suggested for both, but some conflicting. |
| **Ethnicity & nationality** | 56: Sig (Jewish, vs. Arab), 11: Sig (Indian; Malay; Chinese), 53: Sig (China, Vs. AUS) | 52: Sig (Caucasian Vs. others) 43: Sig (Irish Vs. non-Irish)  14: n.s (Malay/Chinese/Indian/other),  32: n.s | 54. Culture and religion and family; 'Building on a strong foundation' |  | Supporting evidence (various- cultural context). |
| **Religion** | 2: Sig. (Students believed in a relationship between taking care of older people and their religious beliefs) , 5: Sig (Religion vs. Secular)  13: n.s. (sec /rel), 29: n.s. (Jewish/ Non) |  |  |  | Limited association for nurses only, not explored in medical. |
| **Family characteristics** | Close relationships:  12: Sig (Close relationship with an elderly relative. Yes Vs No)  12: Sig (Live older family relative. Yes Vs. No) 12: Sig (Have older family members. Yes Vs. No) 13: Sig (Grandparents main caregivers during childhood. Yes Vs. No)  13: n.s (Live with grandparents)  Other:  12: Sig (Parents attitudes to older people: good vs. general/worse) 12: Sig (Only child at home. No Vs. Yes) | Close relationship:  23: n.s (Important relationships)  52: n.s (interaction with grandparent and quality of relationship)  Other:  6: Sig (+) (Perceived influence of friend/family in area) |  | 48: Family Ties | Evidence for a relationship in nurses.  Limited evidence for medical; perceived family and friend influence in area. |
| **Knowledge** | 7: n.s, 56: n.s, 54: n.s, 17: n.s , 40: n.s  40: Sig (+), 55: Sig(+), | 23: n.s, 41 n.s. | 54: Lack of knowledge or ability.  28: Not personally suited.  22: 'Education and nurse educators in elder vs acute care' (feeling unprepared). 35: Feeling underprepared. 49: Do not feel confidence working with older people. | 15: Lack of knowledge (of speciality and exposure to geriatric population | Limited association for both; more qualitative evidence about perceived skills (In nurses). |
| **Positive Attitudes** | To Older people:  3: Sig (+) 16: Sig (+), 33: Sig(+),45: Sig(+), 56: Sig(+), 55: Sig (+), 54: Sig(+), 2:Sig (+), 12 Sig (+), 11: Sig (+), 17: Sig (+), 29: Sig(+), 30:Sig(+) 49: Sig (+) , 42 Sig (+)  51: n.s 22: n.s.  Patients:  44: Sig (+), 2: Sig (+), 13: Sig (+).  Gratitude.  55: Sig (+). | To older people:  3: Sig (+), 41: n.s.  Patients:  14: Sig (+), 23: Sig (+), 23: Sig (+), 52: Sig (+), 32: Sig(+). |  |  | Strong association for a relationship with attitudes to patients and older people (in nursing), a smaller amount of literature on medical but indicative of the same relationship. |
| **Misc. student characteristics** | 13: Grades. n.s.  13: Join school clubs. N.s  13: Paid attention to issues related to older adults. Sig (Yes, Vs. no) |  |  |  | Miscellaneous |
| **CATEGORY 2: COURSE CHARACTERISTICS** | | | | | |
| **Course Characteristics** | Type of course:  11: Sig (Public Vs. private)  29: Sig (College, diploma Vs University)  55: n.s ,11: n.s. ,11: n.s  Integrated vs standalone:  11: n.s, 21: n.s  location:  13: n.s  Nurse educator certified on Gerontological nursing : 11: n.s | Allopathic vs Osteopathic: 52: n.s  Type of geriatric course content: 46: n.s |  |  | No clear associations. Support for public vs private and university vs college in nurses. |
| **CATEGORY 3: EXPERIENCES** | | | | | |
| **Previous experience** | Previous Experience:  8: Sig (+), 55: Sig (+), 12: Sig (+), 29: Sig (+) 11: n.s, 21: n.s, 21: n.s.  Work:  56: Sig (+), 38: Sig (+) 30: Sig (+), 10: n.s, 21:n.s, 49: n.s.  Volunteer: 13: Sig (+)  Amount:  13: Sig (+), 38: Sig (+), 50: Sig (+).  Nursing home experiences: 13: n.s.  Taking related gerontological courses (y/n). 13: n.s. | Previous experience:  46: Sig (+).  14: n.s, 23: n.s. 52: n.s, 18: n.s, 18: n.s, 32: n.s,  Work: 52: n.s.  Voluntary: 52: n.s.  Graduate level entry. 43: Sig (+). | 22: Precollege experiences of elderly |  | Strong support for a positive relationship in nurses. Association not consistently found in medical. |
| **Clinical placements** | Positive clinical placement:  12: Sig (+), 39: Sig (+).  Aspects of placements:  Rating of pedagogical atmosphere in placement 10: Sig (+).  Rating of supervisory relationship. 10: Sig (+)  Usefulness of feedback 39: Sig (+).  Supportiveness of nurse mentors: 39: Sig (+).  Supportiveness of care workers 39: Sig (+).  11: Sig (care home placement, Vs. general or geriatric ward)  Rating of leadership style of manger. 10: N.s.  Rating of premises. 10: n.s.  Role of nursing teacher. 10: N.s. | Perceived influence of positive placement 6: Sig(+)  Self-reported impact of placement on interest 18: Sig(+) | 8: Transformative nature of placements. 20: Influence of clinical placements on career choice.  35: Meeting or challenging expectations. 35: Dispelling Myths. (+) 1: Poor clinical placements  Aspects:  22: Preceptors as role models ( weak for OP care) |  | Moderate support for both medical and nursing students, role of quality highlighted in qualitative literature. |
| **Educational Intervention’s** | 24: Sig (+), 37 Sig (+), 2: Sig (+),  38: n.s, 42: n.s | 32: Sig(+), 41: Sig (+), 43: Sig(+)  9: n.s, 26: n.s, 19: n.s |  | 34 ‘Influence on Specialization’ | Support of effect of several interventions for medical and nursing students. |
| **General experiences** | 8: Sig (recorded previous experience as Positive Vs. negative experience) |  | 54: Positive personal experiences 54: Unconstructive personal experiences.  30: Negative experiences of caring for older people.  25: 'disposition towards elderly' | 48: Amount and Quality  15: Positive experiences with older adults | Support for the association of positive and negative experiences. |
| **CATEGORY 4: CAREER CHARACTERISTICS** | | | | | |
| **Professional development** | 29: Sig (those with higher preference rate opportunity to pursue Clinical Nurse specialists role as influencing factor) |  | 1: Career concerns  50: Negative effect on career  28: Have previously worked in OP (seek new experiences).  31: Future prospect (pursue in future) | 15: ‘Increased demand for geriatric care’ | Lack of perceived professional development negatively associated. Unique for nurses. |
| **Financial and prestige considerations** | Importance of Prestige:  39: Sig(-)  Importance of Finance:  29: n.s | Importance Finance:  18: Sig (-)  Importance of Prestige:  18: Sig (-), 18: Sig (-). | 49: Financial concerns.  1:'Status concerns'  42: Profession (Conditions: pay and staffing)  42: Profession (Culture: stigma and perceived unethical). | 15: Financial concerns  4: Prestige and limited Financial rewards | Evidence for negative association with financial concerns and prestige concerns in nursing medical students. |
| **Lifestyle considerations** |  | 43: Travel rated as important in career. Sig (+)  18: Length of training as a barrier Sig(-)  18: Internal medicine residency (barrier): n.s  18: Lifestyle is an important consideration: n.s 18 Lifestyle issues (barrier) n.s |  |  | Explored in medical only, but limited support. |
| **CATEGORY 5: PATIENT CHARACTERISTICS** | | | | | |
| **Age of patients** | Opportunities to work with older people 39: Sig (+). | Hope practice mostly:  With adults 18: Sig (+)  With children 18: Sig (-)  With seniors 18: Sig (+)  More satisfying working with younger patients 18: Sig (-). |  |  | Positive association for those hoping to work with older patients and those with preferences for working with older people. |
| **Communication difficulties** |  |  | 30: Not being able to communicate or relate to older people.  31: Communication issues.  42: Patient contact (communication) | 4: Interacting with elderly patients | Factor associated with nursing and medical. |
| **Nature of patients illness** |  | 18: I would rather not work with chronically ill patients: Sig (-). 18: Chronicity of patients is a barrier to pursue geriatrics: Sig (-)  18: Find caring for a patient with an acute illness more satisfying (Vs. chronic illness): n.s.  18: Caring for patients with a single well-defined illness: n.s  Cognitive capacity:  Cognitive capacity of patient ( barrier): 18: n.s  Cognitively intact more satisfying 18: n.s | 54: Discomfort with ageing process (depressing and hopeless).  10: Feeling of hopelessness.  28: Lack of clinical recovery | 4: The futility of care  47: Limited capacity to make a difference.  15: Characteristics of the older population (complexity, depressing, boring).  48: Patient responsibility | Factor associated with nursing and medical. |
| **Disposition of patient and family** |  | Family dynamics:  18: Complex family/social issues (barrier): n.s 18: I would rather deal with an older patient with no family members than with an older patient who has family members who need to be informed of the patient’s care: n.s | 54: Positive and negative impressions of older people.  54: Understanding ageing (empathy and the need of elders)  28: characteristics of patients (dislike)  50: Negative view of older people  42: Patient contact (safety) | 4: Unrealistic expectations of patients  48: Perceptions of geriatrics (frustrating, noncompliance) | Evidence for the role of patient dispositions (different concerns for medical and nursing). |
| **CATEGORY 6: WORK CHARACTERISTICS** | | | | | |
| **Complexity** |  | A barrier is my lack of comfort with ambiguity: 18: Sig (-).  18: Complexity of patients (barrier) n.s. |  | 4: The overwhelming nature of managing older patients  47: Being a generalist  4: Difficult ethical dilemmas. | Medical only. |
| **Boring** | Diversity  29: Sig (+, those who rate the current state of gerontological nursing as more diverse) |  | 36: Lack of challenge and boring. 10: Slow-paced and lacking action. 10: Boring, stressful and depressing.  31: Boring  22: Students image of elderly vs reality of work (slow-paced, limited variety, poor prospects)  28: Negative nature of work (boring uninteresting and depressing)  30: Nature of work (boring, physical demands, limited range of tasks- less diversity)  49: Nature of work ( boring, unchallenging and lack of achievement)  28: Less diversity than other areas.  42: Profession (diversity). | 4: Lack of intellectual stimulation.  48: Perceptions of geriatrics (boring) | An overall qualitative theme in nursing and medical students. Pervasive in nursing. |
| **Emotional nature of work**  (Inc. fear and discomfort) | 12: Sig (-) |  | 51: Unpleasant emotions around disabling conditions  28: Fear and discomfort  30: Fear of death and dying.  42: Personal demands (emotional ) | 4: The emotional burden of caring for older people  48: Fear of death and dying (+ associated) | Associated with medical and nursing students. |
| **Control and autonomy** | 29: Nurse powers Sig (+, those who rate nurse powers higher)  29: Expanded case management Sig (+, those who have higher preferences said that increased case powers would influence preferences) |  | Negative:  1:'Fear of being in charge but not in control'  25: Sense of agency  22: 'Influence of power' (lack of).  1: 'A free hand but no one to reach out to'  22: Working alone as a nurse without support.  Positive:  10: 'Independent and autonomous work' (positive aspect of work) |  | A sizable factor for nurses. The factor is complex, with some seemly contradictory elements. |
| **Poor environment** | Positive Ratings of working conditions:  29: Sig (+) |  | Negative:  25: Quality of work life.  10: Unqualified and unengaged staff.  22:'Financial cutbacks' ( impacting care)  49: Work environment  Positive:  10: Harmonious environment ( less stressful) | 47: Understaffed and overworked. | Support as negative factor (more in nursing). |
| **Focus on Quality of life (as a barrier)** |  | 18: Barrier is focus on quality of life Sig (-) | 25: Quality of life of residents  22: ‘Comprehensive view of health care- realities in elder and acute care' | 48: Role of doctors ( to cure) | Support as a negative factor (in both). Shares overlap in with themes related to ‘**nature of patients illness’.** |
| **Positives (long term relationships and rewarding)** | Ability to provide continuity of care 39: Sig (+) |  | 10: Long term relationships.  54: 'Incentives in elder care'  36: Meaningful and enjoyable work.  10: Meaningful and enjoyable work.  51: ‘Developing a value for gerontology’ ( developing relationships, and appreciating complexity) | 48: Perceptions of geriatrics (slower pace, interacting with patients and building relationships) | Positive Association.  Specifically patient interaction in both medical and nursing. |
| **Lack of Procedures** |  | Technical Procedures rated as important in career: 43: Sig (-).  18: Non-procedure-oriented speciality. n.s  18. Think doing a diagnostic procedure (e.g., lumbar puncture) would be more Satisfying than cognitive. n.s |  |  | Limited evidence of a negative association. |
| **Heavy workload** |  |  | 36: Heavy workload  42: Personal demands ( physical) |  | Qualitative evidence as a negative factor. Nursing only. |
| **CATEGORY 7: THEORY OF PLANNED BEHAVIOUR** | | | | | |
| **Attitudes (to behaviour )** | 11: Sig (+) *, 17: Sig (+)* 21: Sig (+), 5: Sig(+)  Behavioural beliefs:  5: Sig (+)  21: Sig (+)  Caring behaviours (attitudes):  17: Sig (-) |  |  |  | Evidence of Positive Association. Nursing only. |
| **Subjective norms** | 5: Sig (+) ,11: Sig(+), 21: Sig(+)  Normative beliefs:  5: Sig (+)  21: Sig(+) |  |  |  | Evidence of Positive Association. Nursing only. |
| **PBC** | 11: Sig (+), [17: n.s.]** 5: n.s,  Control Beliefs:  5: Sig (+) |  |  |  | Mixed evidence for positive association. Nursing only. |
| **Behaviour (job selection)** | 21: Sig (+). |  |  |  | Limited evidence for an association. |

*Included under another factor heading: Attitudes. ** Included under another factor heading: Knowledge.

References

| 1 | Abbey, J., Abbey, B., Bridges, P., Elder, R., Lemcke, P., Liddle, J., & Thornton, R. (2006). Clinical placements in residential aged care facilities: the impact on nursing students' perception of aged care and the effect on career plans. Australian Journal of Advanced Nursing, 23(4), 14-19. | |
| --- | --- | --- |
| 2A | Alsenany, S. (2010). An exploration of the attitudes, knowledge, willingness and future intentions to work with older people among Saudi nursing students in baccalaureate nursing schools in Saudi Arabia. | |
| 2B | Alsenany, S., & Al Saif, A. (2012). Comparison between Saudi and British nursing students toward working with older people. Journal of American Science, 8(7), 316-328. | |
| 3 | Ayoǧlu, F. N., Kulakçı, H., Ayyıldız, T. K., Aslan, G. K., & Veren, F. (2014). Attitudes of Turkish Nursing and Medical Students Toward Elderly People. Journal of Transcultural Nursing, 25(3), 241-248. doi:10.1177/1043659613515527 | |
| 4 | Bagri, A. S., & Tiberius, R. (2010). Medical student perspectives on geriatrics and geriatric education. Journal of the American Geriatrics Society, 58(10), 1994-1999. doi:10.1111/j.1532-5415.2010.03074.x | |
| 5 | Natan, B. M., Danino, S., Freundlich, N., Barda, A., & Mor Yosef, R. (2015). Intention of Nursing Students to Work in Geriatrics. Research in Gerontological Nursing, 8(3), 140-147. doi:10.3928/19404921 -20150219-03 | |
| 6 | Boyle, V., Shulruf, B., & Poole, P. (2014). Influence of gender and other factors on medical student specialty interest. New Zealand Medical Journal, 127(1402), 78-87. | |
| 7 | Briscoe, V. J. (2004). The effects of gerontology nursing teaching methods on nursing student knowledge, attitudes, and desire to work with older adult clients. Ph.D., 127 p-127 p. | |
| 8 | Brown, J., Nolan, M., Davies, S., Nolan, J., & Keady, J. (2008). Transforming students' views of gerontological nursing: Realising the potential of 'enriched' environments of learning and care: A multi-method longitudinal study. International journal of nursing studies, 45(8), 1214-1232. doi:10.1016/j.ijnurstu.2007.07.002 | |
| 9 | Byszewski, A., Bezzina, K., & Latrous, M. (2017). What Kind of Doctor Do You Want to Be? Geriatric Medicine Podcast as a Career Planning Resource. BioMed Research International, 1-6. doi:10.1155/2017/6183148 | |
| 10A | Carlson, E. (2015). Meaningful and enjoyable or boring and depressing? The reasons student nurses give for and against a career in aged care. Journal of Clinical Nursing, 24(3-4), 602-604. doi:10.1111/jocn.12425 | |
| 10B | Carlson, E., & Idvall, E. (2015). Who wants to work with older people? Swedish student nurses' willingness to work in elderly care--a questionnaire study. Nurse education today, 35(7), 849-853. doi:https://dx.doi.org/10.1016/j.nedt.2015.03.002 | |
| 11 | Che, C. C., Chong, M. C., & Hairi, N. N. (2018). What influences student nurses' intention to work with older people? A cross-sectional study. International journal of nursing studies, 85, 61-67. doi:https://dx.doi.org/10.1016/j.ijnurstu.2018.05.007 | |
| 12 | Cheng, M., Cheng, C., Tian, Y., & Fan, X. (2015). Student nurses' motivation to choose gerontological nursing as a career in China: a survey study. Nurse education today, 35(7), 843-848. doi:https://dx.doi.org/10.1016/j.nedt.2015.03.001 | |
| 13 | Chi, M. J., Shyu, M. L., Wang, S. Y., Chuang, H. C., & Chuang, Y. H. (2016). Nursing students’ willingness to care for older adults in Taiwan. Journal of Nursing Scholarship, 48(2), 172-178. | |
| 14 | Chua, M. P., Tan, C. H., Merchant, R., & Soiza, R. L. (2008). Attitudes of first-year medical students in Singapore towards older people and willingness to consider a career in geriatric medicine. Annals of the Academy of Medicine, Singapore, 37(11), 947-951. | |
| 15 | Curran, M. A., Black, M., Depp, C. A., Iglewicz, A., Reichstadt, J., Palinkas, L., & Jeste, D. V. (2015). Perceived barriers and facilitators for an academic career in geriatrics: Medical students’ perspectives. Academic Psychiatry, 39(3), 253-258. doi:10.1007/s40596-014-0208-6 | |
| 16 | Darling, R., Sendir, M., Atav, S., & Buyukyilmaz, F. (2017). Undergraduate nursing students and the elderly: An assessment of attitudes in a Turkish university. Gerontology & Geriatrics Education, 1-12. doi:https://dx.doi.org/10.1080/02701960.2017.1311883 | |
| 17 | de Guzman, A. B., Jimenez, B. C. B., Jocson, K. P., Junio, A. R., Junio, D. E., Jurado, J. B. N., & Justiniano, A. B. F. (2013). Filipino Nursing Students' Behavioral Intentions toward Geriatric Care: A Structural Equation Model (SEM). Educational Gerontology, 39(3), 138-154. | |
|  | |  |
| 18 | Diachun, L. L., Hillier, L. M., & Stolee, P. (2006). Interest in geriatric medicine in Canada: how can we secure a next generation of geriatricians? Journal of the American Geriatrics Society, 54(3), 512-519. doi:10.1111/j.1532-5415.2005.00610.x | |
| 19 | Diachun, L. L., Dumbrell, A. C., Byrne, K., & Esbaugh, J. (2006). But Does It Stick? Evaluating the Durability of Improved Knowledge Following an Undergraduate Experiential Geriatrics Learning Session. Journal of the American Geriatrics Society, 54(4), 696-701. doi:10.1111/j.1532-5415.2006.00656.x | |
| 20 | Duggan, S., Mitchell, E. A., & Moore, K. D. (2013). 'With a bit of tweaking...we could be great'. An exploratory study of the perceptions of students on working with older people in a preregistration BSc (Hons) Nursing course. International Journal of Older People Nursing, 8(3), 207-215. doi:https://dx.doi.org/10.1111/j.1748-3743.2012.00317.x | |
| 21 | Dunkle, S. E., & Hyde, R. S. (1995). Predictors and subsequent decisions of physical therapy and nursing students to work with geriatric clients: an application of the Theory of Reasoned Action. Physical Therapy, 75(7), 614-620. | |
| 22 | Fagerberg, I., Winblad, B., & Ekman, S. L. (2000). Influencing aspects in nursing education on Swedish nursing students' choices of first work area as graduated nurses. Journal of Nursing Education, 39(5), 211-218. | |
| 23 | Fitzgerald, J. T., Wray, L. A., Halter, J. B., Williams, B. C., & Supiano, M. A. (2003). Relating Medical Students' Knowledge, Attitudes, and Experience to an Interest in Geriatric Medicine. The Gerontologist, 43(6), 849-855. doi:10.1093/geront/43.6.849 | |
| 24 | Fox, S. D., & Wold, J. E. (1996). Baccalaureate student gerontological nursing experiences: raising consciousness levels and affecting attitudes. Journal of Nursing Education, 35(8), 348-355. | |
| 25 | Gates, K., Santos, E. J., Nguyen, M., Granovskaya, I., Servidio, A., & Turzanski, M. (2009). Gerontology education initiatives in the health sciences: seeking advice from students in focus group conversations. Perspectives, 33(3), 6-13. | |
| 26 | Gonzales, E., Morrow-Howell, N., & Gilbert, P. (2010). Changing medical students' attitudes toward older adults. Gerontology & Geriatrics Education, 31(3), 220-234. doi:10.1080/02701960.2010.503128 | |
| 27 | Gould, O. N., MacLennan, A., & Dupuis-Blanchard, S. (2012). Career preferences of nursing students. Canadian Journal on Aging, 31(4), 471-482. doi:10.1017/S0714980812000359 | |
| 28A | Happell, B. (1999). When I grow up I want to be a...? Where undergraduate student nurses want to work after graduation. Journal of advanced nursing, 29(2), 499-505. | |
| 28B | Happell, B. (2002a). Nursing home employment for nursing students: valuable experience or a harsh deterrent? Journal of advanced nursing, 39(6), 529-536. | |
| 28C | Happell, B. (2002b). The role of nursing education in the perpetuation of inequality. Nurse education today, 22(8), 632-640. | |
| 28D | Happell, B., & Brooker, J. (2001). Who will look after my grandmother? Attitudes of student nurses toward the care of older adults. Journal of gerontological nursing, 27(12), 12-17. | |
| 29 | Haron, Y., Levy, S., Albagli, M., Rotstein, R., & Riba, S. (2013). Why do nursing students not want to work in geriatric care? A national questionnaire survey. International journal of nursing studies, 50(11), 1558-1565. doi:10.1016/j.ijnurstu.2013.03.012 | |
| 30 | Henderson, J., Xiao, L., Siegloff, L., Kelton, M., & Paterson, J. (2008). 'Older people have lived their lives': First year nursing students' attitudes towards older people. Contemporary Nurse, 30(1), 32-45. doi:10.5172/conu.673.30.1.32 | |
| 31 | Herdman, E. (2002). Challenging the discourses of nursing ageism. International journal of nursing studies, 39(1), 105-114. | |
| 32 | Hughes, N. J., Soiza, R. L., Chua, M., Hoyle, G. E., MacDonald, A., Primrose, W. R., & Seymour, D. G. (2008). Medical student attitudes toward older people and willingness to consider a career in geriatric medicine. Journal of the American Geriatrics Society, 56(2), 334-338. doi:10.1111/j.1532-5415.2007.01552.x | |
| 33 | Hweidi, I. M., & Al-Obeisat, S. M. (2006). Jordanian nursing students' attitudes toward the elderly. Nurse education today, 26(1), 23-30. | |
| 34 | Jefferson, A. L., Cantwell, N. G., Byerly, L. K., & Morhardt, D. (2012). Medical student education program in Alzheimer's disease: the PAIRS Program. BMC medical education, 12, 80. doi:https://dx.doi.org/10.1186/1472-6920-12-80 | |
| 35 | King, B. J., Roberts, T. J., & Bowers, B. J. (2013). Nursing student attitudes toward and preferences for working with older adults. Gerontology & Geriatrics Education, 34(3), 272-291. doi:10.1080/02701960.2012.718012 | |
| 36 | Kloster, T., Høie, M., & Skår, R. (2007). Nursing students' career preferences: A Norwegian study. Journal of advanced nursing, 59(2), 155-162. doi:10.1111/j.1365-2648.2007.04276.x | |
| 37 | Koehler, A. R., Davies, S., Smith, L. R., Hooks, T., Schanke, H., Loeffler, A., . . . Ratzlaff, N. (2016). Impact of a stand-alone course in gerontological nursing on undergraduate nursing students' perceptions of working with older adults: A Quasi-experimental study. Nurse education today, 46, 17-23. doi:10.1016/j.nedt.2016.06.015 | |
| 38A | Koskinen, S. (2016). Nursing students and older people nursing. Towards a future career. | |
| 38B | Koskinen, S., Hupli, M., Katajisto, J., & Salminen, L. (2012). Graduating Finnish nurse students' interest in gerontological nursing--a survey study. Nurse education today, 32(4), 356-360. doi:https://dx.doi.org/10.1016/j.nedt.2011.05.015 | |
| 39 | Lea, E., Mason, R., Eccleston, C., & Robinson, A. (2016). Aspects of nursing student placements associated with perceived likelihood of working in residential aged care. Journal of Clinical Nursing, 25(5-6), 715-724. doi:10.1111/jocn.13018 | |
| 40 | Lee, A. C. K., Wong, A. K. P., & Loh, E. K. Y. (2006). Score in the Palmore’s Aging Quiz, knowledge of community resources and working preferences of undergraduate nursing students toward the elderly in Hong Kong. Nurse education today, 26(4), 269-276. | |
| 41 | Lu, W.-H., Hoffman, K. G., Hosokawa, M. C., Gray, M. P., & Zweig, S. C. (2010). First year medical students' knowledge, attitudes, and interest in geriatric medicine. Educational Gerontology, 36(8), 687-701. | |
| 42 | McKenzie, E. L., & Brown, P. M. (2014). Nursing students' intentions to work in dementia care: Influence of age, ageism, and perceived barriers. Educational Gerontology, 40(8), 618-633. doi:10.1080/03601277.2013.863545 | |
| 43 | Ni Chroinin, D., Cronin, E., Cullen, W., O'Shea, D., Steele, M., Bury, G., & Kyne, L. (2013). Would you be a geriatrician? Student career preferences and attitudes to a career in geriatric medicine. Age & Ageing, 42(5), 654-657. doi:https://dx.doi.org/10.1093/ageing/aft093 | |
| 44 | Pan, I. J., Edwards, H., & Chang, A. (2009). Taiwanese Nursing Students' Attitudes Toward Older People. Journal of gerontological nursing, 1-6. doi:https://dx.doi.org/10.3928/00989134-20090903-01 | |
| 45 | Rathnayake, S., Athukorala, Y., & Siop, S. (2016). Attitudes toward and willingness to work with older people among undergraduate nursing students in a public university in Sri Lanka: A cross sectional study. Nurse Educ Today, 36, 439-444. doi:10.1016/j.nedt.2015.10.007 | |
| 46 | Robbins, T. D., Crocker-Buque, T., Forrester-Paton, C., Cantlay, A., Gladman, J. R. F., & Gordon, A. L. (2011). Geriatrics is rewarding but lacks earning potential and prestige: responses from the national medical student survey of attitudes to and perceptions of geriatric medicine. Age & Ageing, 40(3), 405-408. doi:ageing/afr033 | |
| 47 | Samra, R. (2013). Medical students; and doctors attitudes toward older patients and their care: what do we known and where do we go from here? , Ph.D. | |
| 48 | Schigelone, A. S., & Ingersoll-Dayton, B. (2004). Some of my Best Friends are Old: A Qualitative Exploration of Medical Students' Interest in Geriatrics. Educational Gerontology, 30(8), 643-661. doi:10.1080/03601270490483887 | |
| 49 | Shen, J., & Xiao, L. D. (2012). Factors affecting nursing students' intention to work with older people in China. Nurse education today, 32(3), 219-223. doi:10.1016/j.nedt.2011.03.016 | |
| 50 | Stevens, J. A. (2011). Student nurses’ career preferences for working with older people: A replicated longitudinal survey. International journal of nursing studies, 48(8), 944-951. doi:10.1016/j.ijnurstu.2011.01.004 | |
| 51 | Swanlund, S., & Kujath, A. (2012). Attitudes of baccalaureate nursing students toward older adults: a pilot study. Nursing Education Perspectives, 33(3), 181-183. | |
| 52 | Voogt, S. J., Mickus, M., Santiago, O., & Herman, S. E. (2008). Attitudes, experiences, and interest in geriatrics of first-year allopathic and osteopathic medical students. Journal of the American Geriatrics Society, 56(2), 339-344. doi:10.1111/j.1532-5415.2007.01541.x | |
| 53 | Xiao, L. D., Shen, J., & Paterson, J. (2013). Cross-cultural comparison of attitudes and preferences for care of the elderly among Australian and Chinese nursing students. Journal of Transcultural Nursing, 24(4), 408-416. doi:10.1177/1043659613493329 | |
| 54 | Zakari, N. M. A. (2005). Attitudes toward the elderly and knowledge of aging as correlates to the willingness and intention to work with elderly among Saudi nursing students. Ph.D., 236 p-236 p. | |
| 55 | Zhang, S., Liu, Y.-h., Zhang, H.-f., Meng, L.-n., & Liu, P.-x. (2016). Determinants of undergraduate nursing students' care willingness towards the elderly in China: Attitudes, gratitude and knowledge. Nurse education today, 43, 28-33. | |
| 56 | Zisberg, A. P. R. N., Topaz, M. M. A. R. N., & Band-Wintershtein, T. P. (2015). Cultural- and Educational-Level Differences in Students Knowledge, Attitudes, and Preferences for Working With Older Adults: An Israeli Perspective. Journal of Transcultural Nursing, 26(2), 193. | |
